# Relative Validity of Food Intake in Each Meal Type and Overall Food Intake Derived Using the Meal-Based Diet History Questionnaire against the 4-Day Weighed Dietary Record in Japanese Adults

**DOI:** 10.3390/nu14153193

**Published:** 2022-08-04

**Authors:** Kentaro Murakami, Nana Shinozaki, Nana Kimoto, Shizuko Masayasu, Satoshi Sasaki

**Affiliations:** 1 Department of Social and Preventive Epidemiology, School of Public Health, University of Tokyo, Tokyo 113-0033, Japan; 2 Ikurien-Naka, Ibaraki 311-0105, Japan

**Keywords:** diet, meal, snack, food, questionnaire, dietary record, validity, Japan

## Abstract

We examined the relative validity of food intake for each meal type (breakfast, lunch, dinner, and snacks) and overall food intake obtained through the Meal-based Diet History Questionnaire (MDHQ). In total, 222 Japanese adults (111 for each sex) aged 30–76 years completed the web version of the MDHQ and then the 4-non-consecutive-day weighed dietary record (DR). The number of major food groups (*n* = 24 in total) for which no statistically significant difference was observed between median intakes estimated using the DR and MDHQ ranged from 8 (snacks) to 12 (dinner) among women, and from 8 (breakfast) to 13 (lunch) among men. The median values of the Spearman’s correlation coefficients between the MDHQ and DR estimates ranged from 0.28 (dinner) to 0.54 (breakfast) among women, and from 0.24 (dinner) and 0.60 (breakfast) among men. Bland–Altman analyses generally showed wide limits of agreement and proportional bias. Similar results were obtained using the paper version of the MDHQ, which was completed after conducting the DR. In conclusion, the MDHQ has a satisfactory ability to estimate median intake and rank individuals according to consumption for many food groups, despite a limited ability to estimate food group intakes on an individual level.

## 1. Introduction

Suboptimal dietary intake is widely acknowledged as a major risk factor for morbidity and premature death, and improving the quality of diet is now a global priority [1]. An accurate assessment of habitual dietary intake is a prerequisite for uncovering the diet–disease relationships and developing appropriate advice to support favorable changes in dietary behaviors [2]. Traditionally, nutritional epidemiologic research has examined the associations between health outcomes and the intake of individual nutrients or foods, with a gradual shift in the past several decades to examine the associations between health outcomes and overall dietary patterns [3]. More recently, an increasing number of studies have evaluated dietary intake in specific eating occasions or meal patterns [4,5,6]. Examining dietary patterns and intake at each meal level to assess the overall diet may be more pertinent when considering the synergies and interactions during digestion and metabolism [7]. A limited number of studies have suggested that the amount and type of food intake, as well as the circadian timing of food intake, are associated with health status [8], such as obesity [9], metabolic syndrome [10], hyperglycemia [11], non-alcoholic fatty liver disease [9], and muscle strength [12]. Information on diet–health relationships at each meal level would be more relevant for formulating meaningful dietary guidelines and public health messages, as well as for developing effective intervention strategies for promoting healthy eating habits.

Unfortunately, research in this area is constrained by the fact that the primary method of dietary assessment currently employed in most cross-sectional and prospective cohort studies is the food frequency questionnaire, which precludes an informed evaluation of the timing of dietary intake and meal-specific dietary intake [13]. This type of information can be derived using more detailed dietary assessment methods, such as dietary record (DR) and 24-h dietary recall [4]. However, when using these methods, the collection of dietary data for multiple days is essential for the assessment of habitual intake at the individual level, but it is not always feasible because of its expensive and burdensome nature [14], despite the advancement of technology [15]. Taken together, the development and validation of a dedicated fit-for-purpose methodology for collecting data on meal patterns and time of day of dietary intake, which is also inexpensive to administer and has a low participant burden, are required to efficiently improve this research field [4]. In this context, we recently developed the Meal-based Diet History Questionnaire (MDHQ), a self-administered questionnaire designed to estimate the dietary intake for each meal type (breakfast, lunch, dinner, and snacks) separately [16,17].

This study aimed to examine the relative validity of food intake for each meal type and overall food intake obtained through the MDHQ against the 4-day DR.

## 2. Materials and Methods

### 2.1. Study Procedure and Participants

This cross-sectional study was based on the data collected from 14 (of the 47) prefectures between August and October 2021. Recruitment of participants and data collection were conducted by our research dietitians (*n* = 66) with expertise in collecting DR data [18,19]. First, healthy women aged 30–69 years who were willing to participate and were living with their husbands were recruited for this study. For each prefecture, two women from each 10-year age category (30–39, 40–49, 50–59, and 60–69 years) were selected. Their husbands were then recruited (irrespective of age), resulting in 112 individuals invited for each sex. The sample size was determined primarily based on the recommendation made by Cade et al. [14]. To minimize the dropout rate, the potential participants were restricted to individuals who had full understanding of the procedure, were willing to endure the heavy burden of the survey, and showed willingness to complete the entire survey. Meanwhile, dietitians, individuals living with a dietitian, those who had received dietary counseling from a doctor or dietitian, those taking insulin treatment for diabetes, those undergoing dialysis treatment, those without sufficient Internet access, those who had difficulty answering the web-based questionnaires, and pregnant or lactating women were excluded. Those who participated in the study as a couple (one woman and one men) were permitted. Due to the use of the snowball sampling procedure, the number of individuals approached for this study and the number of individuals excluded from this study were not formally recorded.

**Figure 1:**
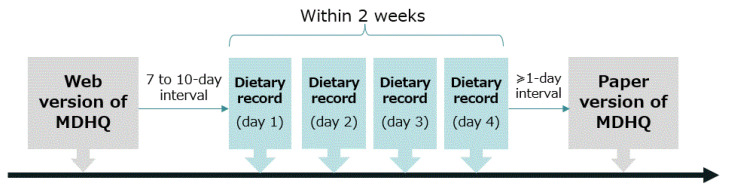
Study schedule. MDHQ, Meal-based Diet History Questionnaire; DR, dietary record.

The study schedule is shown in Figure 1. Each participant was asked to answer the web version of the MDHQ (web MDHQ). After an interval of 7 to 10 days (to ensure the completion of the web MDHQ), a 4-non-consecutive-day weighed DR was conducted for 2 weeks. Finally, after an interval of at least 1 day, the paper version of the MDHQ (paper MDHQ) was answered. We designed this schedule because the main purpose of this study was to evaluate the validity of the web MDHQ; thus, a web MDHQ survey was performed prior to the conduct of DR. A total of 111 women aged 30–69 years and 111 men aged 30–76 years completed the study.

The study was conducted in accordance with the guidelines of the Declaration of Helsinki, and all procedures involving humans were approved by the Ethics Committee of the University of Tokyo Faculty of Medicine (protocol code: 2020326NI; date of approval: 29 January 2021). Written informed consent was obtained from all participants.

### 2.2. Meal-Based Diet History Questionnaire

Details of the MDHQ have been published elsewhere [16,17]. Briefly, the MDHQ is a self-administered questionnaire designed to estimate dietary intake in the previous month for each meal type (breakfast, morning snack, lunch, afternoon snack, dinner, and night snack).

The MDHQ comprises three parts. Part 1 of the MDHQ includes quantitative questions on the consumption frequency of generic food groups (Tier 1 food groups) for each meal type, with potential answers of 0–7 days/week. An a priori decision was made not to collect information on portion sizes using the MDHQ (except for alcoholic beverages, for which the overall consumption frequency and portion size were assessed in Part 2). This decision was based on our previous observation that the Brief-type Diet History Questionnaire (BDHQ), which assesses the consumption frequency of 58 food items but does not collect information on portion sizes and applies fixed portion sizes for dietary intake calculation, had a similar efficacy in estimating the food and nutrient intake as the Diet History Questionnaire (DHQ), which assesses not only the consumption frequency, but also the portion size of 150 food items [20,21,22]. The limited usefulness of portion size information has also been supported by several previous studies [23,24]. For each of the Tier 1 food groups, sex-specific and meal-type specific fixed portion size was defined as the mean of the intake value of the respective Tier 1 food group in independent 16-day weighed DR data previously collected from 121 Japanese women and 121 Japanese men, comprising 206,837 food item entries [16].

Part 2 of the MDHQ includes questions on the relative consumption frequency of sub-food groups (Tier 2 food group) within one of the generic food groups (Tier 1 food group), with possible answers of “always, often, sometimes, rarely, and never.” By combining the information derived from Parts 1 and 2, the number of foods that can be estimated efficiently can be increased, but within a limited number of questions.

Part 3 of the MDHQ asks about the general eating behaviors, including the relative consumption frequency of brown rice and wholegrain bread, followed by assessment of the baseline characteristics (sex, age, body height, body weight, education level, and current smoking status). All the food groups included in the MDHQ (see supplementary material Table S1) were determined based on the 16-day weighed DR data [16].

In the present study, two modes of MDHQ, which are identical in terms of content, were used: web MDHQ and paper MDHQ. The web MDHQ was prepared using Google Forms. Each question was answered by each participant, and no blanks were permitted. All responses to the web MDHQ automatically allocated into a spreadsheet format were downloaded from Google Drive. The paper MDHQ used in this study was an A4 21-page questionnaire. Responses to all questions were checked by the research dietitians and staff at the study center. If any responses were missing, the participants were asked to answer the questions again in person or by phone. All answers in the paper MDHQ were manually entered into a spreadsheet in duplicate, and any disagreement was checked and corrected.

Data obtained using the web MDHQ and paper MDHQ were converted to a dataset suitable for dietary intake calculation. Estimates of food group intake were calculated using ad hoc calculation algorithms, the details of which are available elsewhere [16]. Briefly, the intake of each Tier 1 food group for each meal type was calculated as the consumption frequency (from Part 1) multiplied by sex-specific and meal-type-specific portion size, as well as by a weighting factor determined by age using the estimated energy requirement [25]. The intake of each Tier 2 food group within the respective Tier 1 food group for each meal type was calculated based on the intake of the respective Tier 1 food group, meal-type-specific standard proportion of Tier 2 food groups (determined during the development process using the 16-day weighed DR data), and weighting factors created based on the data of relative consumption frequency (from Part 2). The overall intake was calculated as the sum of the intake of each meal type. For each alcoholic beverage, the overall intake was calculated as the product of consumption frequency and portion size, and then disaggregated into each meal type using the responses to questions related to alcoholic beverages in Part 1.

### 2.3. Weighed Dietary Record

The 4-non-consecutive-day weighed DR was selected as the reference method in this validation study. Each recording period consisted of three weekdays (Monday–Friday, except for national holidays) and one weekend day (Saturday, Sunday, or national holidays). For each couple, a recording day was allocated within 2 weeks by research dietitians. Each couple was provided with recording sheets and a digital scale (KS-274, Tanita, Japan; ±2 g precision for 0–500 g and ±3 g precision for 500–2000 g). After receiving written and verbal instructions from the assigned research dietitian, as well as an example of a completed diary sheet, each participant was requested to document and weigh all consumed foods and drinks, both inside and outside of their homes, on each recording day. On certain occasions when weighing was difficult to carry out (e.g., dining out), they were instructed to document as much information as possible, including the brand name of the food and the consumed portion size (based on typical household measures), as well as the details of the leftovers.

The recording sheets used in each survey day were submitted directly to the research dietitian after the survey was completed, who then reviewed the forms and, whenever necessary, sought additional information or modified the record via phone or in-person interview. All collected records were then reviewed by the research dietitians and trained staff at the study center. In accordance with a standardized procedure, the portion sizes estimated using household measures were converted into weights, and the individual food items were coded based on the 2015 version of the Standard Tables of Food Composition in Japan [26]. A total of 1297 food codes were used in the DR. All food codes were classified into one of the Tier 2 or 1 food groups in the MDHQ.

The structure of the food diary sheet used was based on a typical Japanese eating pattern, which comprised breakfast, lunch, dinner, and snacks; these meal types were prescribed in the diary. Thus, the name of the meal type used in the present analysis was based on this classification. For each participant, the estimated intake of individual food groups was calculated for each meal type. Foods expressed in the dry-weight state but consumed after cooking were corrected to represent the amount as consumed. The overall intake was calculated as the sum of the intake from each meal type. For all dietary variables, the mean daily values within the 4-day period were used for each individual.

### 2.4. Statistical Analysis

Statistical analyses were performed using the SAS statistical software (version 9.4; SAS Institute Inc., Cary, NC, USA). A two-tailed *p* value of <0.05 was considered significant. The dietary variables examined in this study included intake of Tier 1 and 2 food groups; the investigation of other variables (e.g., energy and nutrients) is under preparation and will be presented elsewhere. The amounts of snacks consumed were combined for analysis due to their relatively low intake in both methods. Analyses were stratified by sex and conducted to examine the overall intake and intake for each meal type (breakfast, lunch, dinner, and snacks). For the Tier 2 food groups, only the overall intake was considered. For each dietary assessment method, total daily energy intake was calculated using the 2015 version of the Standard Tables of Food Composition in Japan [26].

All dietary data were expressed as medians (25th and 75th percentiles) and means (standard deviations). To assess the estimation ability at the group level, the median values of intake derived from the MDHQ were compared with those derived from the DR using the Wilcoxon signed-rank test. The mean values were also compared using a paired *t*-test. The Spearman’s correlation coefficients between MDHQ and DR estimates were used to assess the ability of the MDHQ to rank individuals in a population. In addition, agreement of the dietary variables between the MDHQ and DR was assessed using the Bland–Altman analysis [27]. Linear regression analysis was also used to examine the proportional bias between the MDHQ and DR [28]. Identical analyses were conducted to assess the web MDHQ and paper MDHQ. The findings on the former are provided in the Results section, whereas those on the latter are provided as supplemental materials.

## 3. Results

This study included 111 women and 111 men aged 30–69 years and 30–76 years, respectively (Table 1). The mean body mass index values (kg/m^2^) were 22.7 (standard deviation: 3.3) for women and 23.8 (standard deviation: 3.6) for men. For both women and men, mean total energy intake derived from the DR was significantly (*p* < 0.001) higher than that derived from either the web MDHQ or the paper MDHQ.

### 3.1. Median Estimation of Tier 1 Food Groups

The median estimates of daily intake of 24 Tier 1 food groups derived from the DR and web MDHQ are shown in Table 2 for women and Table 3 for men, according to meal type. Among women, the number of food groups (and % of the total number of food groups) for which no statistically significant difference was observed between median intakes estimated using the DR and web MDHQ was 11 (46%) for breakfast, 11 (46%) for lunch, 12 (50%) for dinner, 8 (33%) for snacks, and 10 (42%) for the overall diet. The corresponding number among men was 8 (33%) for breakfast, 13 (54%) for lunch, 12 (50%) for dinner, 12 (50%) for snacks, and 9 (38%) for the overall diet. When comparisons were made using the mean values, the results were similar (Table S2 for women and Table S3 for men).

### 3.2. Spearman’s Correlation of Tier 1 Food Groups

The Spearman’s correlation coefficients between the estimates of daily intake of the 24 Tier 1 food groups derived from the DR and those derived from the web MDHQ are shown in Table 4, according to meal type. For women, the median values of the Spearman’s correlation coefficients (25th and 75th percentiles) were 0.54 (0.38–0.62) for breakfast, 0.30 (0.21–0.42) for lunch, 0.28 (0.20–0.49) for dinner, 0.47 (0.35–0.54) for snacks, and 0.47 (0.42–0.59) for the overall diet. The corresponding values for men were 0.60 (0.46–0.67) for breakfast, 0.34 (0.26–0.41) for lunch, 0.24 (0.15–0.38) for dinner, 0.39 (0.33–0.45) for snacks, and 0.49 (0.35–0.59) for the overall diet.

### 3.3. Bland–Altman Analysis of Tier 1 Food Groups

**Figure 2:**
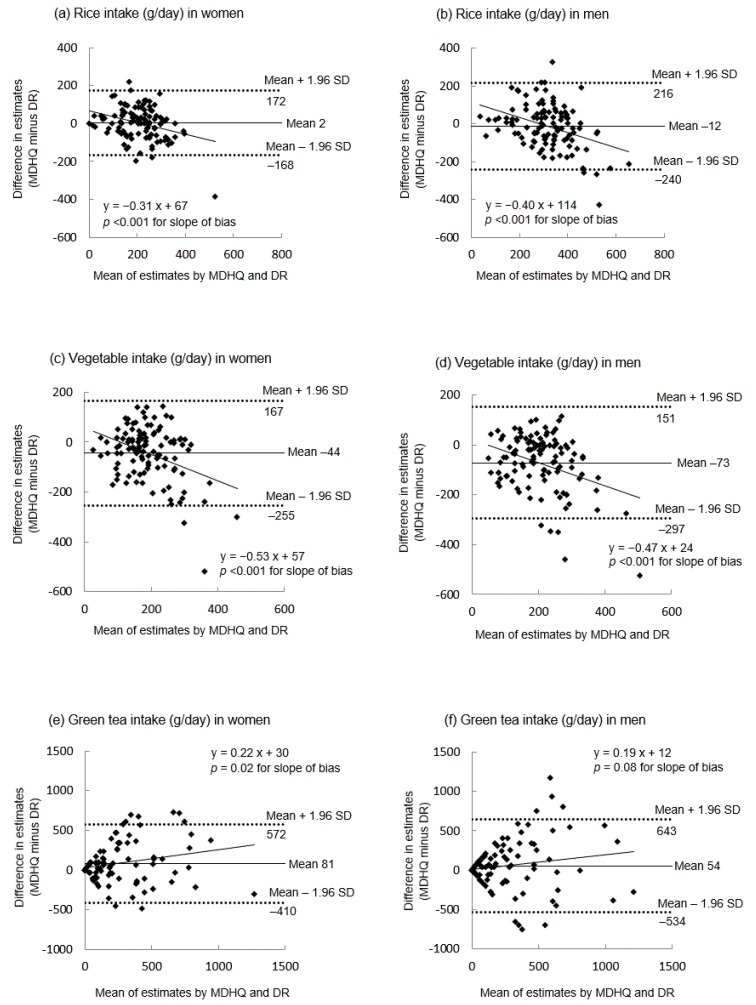
Bland–Altman plots assessing the agreement between estimates of overall intakes of rice (**a**,**b**), vegetables (**c**,**d**), and green tea (**e**,**f**) derived from the 4-day weighed dietary record (DR) and those derived from the web version of the Meal-based Diet History Questionnaire (MDHQ) in 111 Japanese women and 111 Japanese men. SD, standard deviation.

Based on the results of Bland–Altman analysis, the mean differences between estimates of daily intake of the 24 Tier 1 food groups derived from the DR and those derived from the web MDHQ (i.e., web MDHQ minus DR) were −8 to 54 g for breakfast, −19 to 57 g for lunch, −25 to 53 g for dinner, −16 to 45 g for snacks, and −44 to 209 g for the overall diet in women (Table S4). The corresponding values in men (Table S5) were −12 to 49 g for breakfast, −39 to 66 g for lunch, −46 to 77 g for dinner, −19 to 25 g for snacks, and −73 to 212 g for the overall diet. Figure 2 shows the Bland–Altman plots for overall daily intake of rice (mean differences: 2 g for women and −12 g for men), vegetables (−44 and −73 g, respectively), and green tea (81 and 54 g, respectively) as examples of acceptable estimation, underestimation, and overestimation by the web MDHQ, respectively. Regardless of the food group, sex, and meal type, the limits of agreement (mean difference ± 1.96 standard deviation of the difference) were generally wide, indicating poor agreement at the individual level (Figure 2 and Tables S4 and S5). In many cases, the slope of bias was significant, but solid foods tended to be underestimated by the web MDHQ as the average intake increased, while beverages tended to be overestimated by the web MDHQ as the average intake increased (Figure 2 and Tables S4 and S5).

### 3.4. Estimation of the Tier 2 Food Groups

The same analyses were conducted for the overall intake of Tier 2 food groups. Table 5 shows the median estimates of daily intake of 87 Tier 2 food groups derived using the DR and web MDHQ. The numbers of Tier 2 food groups showing no significant differences between the web MDHQ and DR were 43 (49%) for women and 41 (47%) for men. When comparisons were made using the mean values, the results were similar (Table S6).

The Spearman’s correlation coefficients between estimates of daily intake of 87 Tier 2 food groups derived from the DR and those derived from the web MDHQ are also shown in Table 5. The median values of the Spearman’s correlation coefficients (25th and 75th percentiles) were 0.25 (0.16–0.36) for women and 0.27 (0.15–0.35) for men.

Based on the Bland–Altman analysis (Table S6), the mean differences between the estimates of daily intake of 87 Tier 2 food groups derived from the DR and web MDHQ (i.e., web MDHQ minus DR) were −29 g (yogurt) to 18 g (full-fat milk) for women, and −27 g (white rice) to 33 g (beer) for men. Regardless of food group and sex, the limits of agreement (mean difference ± 1.96 standard deviation of the difference) were generally wide, indicating poor agreement at the individual level (Table S6). In many cases, the slope of bias was significant, showing a general tendency of underestimation by the web MDHQ as the average intake increased (Table S6).

### 3.5. Results on the Paper Version of the Meal-Based Diet History Questionnaire

Identical analyses of the paper MDHQ were conducted in terms of both Tier 1 food groups (Table S7 for median estimation, Table S8 for mean estimation, Table S9 for Spearman’s correlation, Table S10 for Bland–Altman analysis among women, and Table S11 for Bland–Altman analysis among men) and Tier 2 food groups (Table S12 for all analyses). The results for the paper MDHQ were generally similar to those for the web MDHQ, except for somewhat high Spearman’s correlation coefficients between the paper MDHQ and the DR.

## 4. Discussion

To our knowledge, this study was the first to examine the usefulness of a self-administered dietary assessment questionnaire specifically designed to estimate the dietary intake for each meal type separately (i.e., MDHQ). Overall, the present analysis suggests that the web and paper versions of the MDHQ showed reasonable relative validity in terms of food intake against the 4-day weighed DR. The MDHQ had a satisfactory ability to estimate median intake and rank individuals according to consumption for many food groups, despite a limited ability to estimate food group intakes at the individual level.

To estimate the overall intake of major food groups (Tier 1 food groups in this study), the present findings are reasonable in comparison with the results of previous relative validation analyses of the DHQ and BDHQ [20]. No significant median differences were noted between the DHQ and 16-day DR in 44% and 41% of food groups among 92 women and 92 men, respectively; moreover, no significant median differences were observed between the BDHQ and 16-day DR in 52% and 55% of food groups among 92 women and 92 men, respectively [20]. In terms of ranking individuals according to their overall intake, the median Spearman’s correlation coefficient in this study was comparable with that for the DHQ (0.43 for women and 0.44 for men) and BDHQ (0.44 for women and 0.48 for men) [20]. These findings are not uncommon in other dietary assessment questionnaires in Japan [29,30,31]. Taken together, at least for the overall intake of major food groups, the present study suggests that the MDHQ’s ability is not inferior to that of the DHQ and BDHQ, the most widely used dietary assessment questionnaires in Japan.

In general, we found that the level of concordance between intake of Tier 1 food groups derived from the MDHQ and 4-day DR was similar across all meal types based on the median intake estimation and impressions from Bland–Altman analysis, but the ability to rank individuals according to intake level was higher for breakfast and snacks than for lunch and dinner. This finding may be due to the large between-person variability of food intake patterns at breakfast and snacks compared with that at lunch and dinner [6,19,32]. Alternatively, this may reflect the complex nature of lunch and dinner in terms of food consumption patterns compared with breakfast and snacks [33,34]. To support this finding, the median value of Pearson’s correlation coefficients between energy intake from 12 food groups estimated using a food frequency questionnaire and a 7-day DR in a small study of Japanese adolescent girls (*n* = 63) was higher at breakfast (0.71) than that at lunch (0.38) and dinner (0.44); this questionnaire was not designed to assess the snack intake [35]. Similar results were also observed in a small sample of Japanese adults (29 men and 60 women) [36]. Overall, the present findings support the appropriateness of the MDHQ for assessing meal-specific dietary intake.

Nevertheless, the efficacy of MDHQ varied substantially depending on the Tier 1 food groups. For example, its ranking ability (assessed by Spearman’s correlation coefficient) was somewhat high in certain food groups such as rice, miso soup, fruit, dairy products, alcoholic beverages, green tea, barley tea, and coffee. This may be because variations in the intake of these foods are largely determined based on the consumption frequency, which was assessed in Part 1 of the MDHQ. Considering that the MDHQ does not collect any information on portion sizes, this finding may also suggest that the portion size of these foods is occasion specific (or meal type specific), rather than person specific. This speculation may be reasonable because these foods are all central components of the Japanese dietary patterns [32,37,38]. On the contrary, the ranking ability was somewhat low in other food groups such as noodles, potatoes, fish and shellfish, and meat, suggesting that variations in intake of these foods are largely determined by portion size (in addition to consumption frequency), and that the accurate estimation of consumption frequency is challenging for these foods. This may again be reasonable because, in Japan, these foods are usually consumed in combination with a variety of foods (e.g., composite dishes, soup, and salad), in a very small amount (e.g., toppings for noodles), or both, resulting in the variations of portion sizes (as well as difficulty in the accurate estimation). Nevertheless, simply collecting information on a single portion size may not substantially improve the estimation ability of these foods, considering that the relative validity of the DHQ (which allows collection of the portion size information) was not superior to that of the BDHQ (which does not include collection of portion size information) [20,21,22].

The present study also showed that the efficacy of MDHQ varied substantially depending on the Tier 2 food groups. For example, its ranking ability (as assessed by the Spearman correlation coefficient) was somewhat high in certain food groups such as white rice, white bread, natto (fermented soybeans), bananas, low-fat milk, yogurt, high-fat milk, beer, and shochu (Japanese distilled beverages). As we are unaware of validation studies conducted at the food item level, the present study provides valuable insights into the selection and design of dietary surveys in Japan. As the primary measure in dietary assessment is food intake, it is important to understand which foods and meal types are difficult to measure to improve the efficacy of MDHQ, as well as other dietary assessment methods.

Furthermore, irrespective of sex, meal type, and food group, Bland-Altman plots showed poor agreement between the MDHQ and DR at the individual level, although the mean difference was generally small. This is consistent with previous studies on relative validity of the DHQ and BDHQ [20]. In the MDHQ, the use of the fixed portion sizes during dietary intake calculation might at least partly explain poor agreement at the individual level. In any case, the absolute values of food intakes derived from the MDHQ should be interpreted with considerable caution at the individual level.

In this study, the findings for the web MDHQ were generally similar to those for the paper MDHQ, although the Spearman’s correlation coefficients with DR were somewhat high for the paper MDHQ compared with that for the web MDHQ. This is not surprising given that the paper MDHQ was completed after conducting the DR, while the web MDHQ was completed before conducting the DR. Online questionnaires are preferred for administration and processing because they are inexpensive. In real-world settings, not all study participants may be willing to complete the online questionnaires. Thus, a direct comparison between the web and paper versions of the MDHQ is warranted to assess the comparability or compatibility of these two modes.

The present study has several limitations. First, although the survey was conducted in diverse regions (14 of 47 prefectures), the present population was not a nationally representative sample of the Japanese population. As volunteers, the participants may have been biased toward greater health consciousness, higher socioeconomic status, or both. For example, the education level in the present population was higher than that in a national representative sample of women (55.9% completed junior high school or high school, 27.6% completed college or technical school, and 15.6% completed a university degree or higher) and men (52.9%, 12.9%, and 33.7%, respectively) [39]. Meanwhile, the prevalence of current smokers and mean (standard deviation) values of body height, body weight, and body mass index in the present participants were similar to those in a nationally representative sample (women: 7.6%, 154.3 (6.7) cm, 53.6 (9.2) kg, and 22.5 (3.7) kg/m^2^, respectively; men: 27.1%, 167.7 (6.9) cm, 67.4 (12.0) kg, and 23.9 (3.6) kg/m^2^, respectively) [40]. Ideally, further validation should be conducted using a more representative sample.

Second, weighed DR was used as a reference method; however, weighed DR is also susceptible to measurement errors due to the erroneous recording and potential changes in eating behavior [14]. However, weighed DR is the first method of choice for validating the dietary assessment questionnaires because the errors in weighed DR are thought to be less correlated with those in dietary assessment questionnaires compared with the errors in 24-h dietary recall or other instruments that rely on memory [14]. Additionally, although the dietary recording period was set to four days, this duration might not be sufficient for capturing estimates of habitual intake (particularly for certain Tier 2 food groups). Considering that increasing the number of recording days in the reference method improves the apparent validity of a dietary assessment questionnaire [14,41], efforts to increase the duration of recording in the reference method would be important in future validation studies.

Finally, the data collection was conducted over a certain period (between August and October 2021; late summer and early autumn in Japan). Considering the seasonal differences in the intake of at least some food groups in Japanese adults [42,43,44], and that the MDHQ only assessed the dietary habits during the previous month, the present data collection would have been conducted throughout the year. However, results of our previous validation study of the DHQ and BDHQ suggested that a single administration of a questionnaire assessing the dietary habits during the previous month may reasonably capture the habitual dietary intake over a longer period (i.e., one year) [20,21,22,45]. There is no strong reason to consider that the MDHQ is an exception in this regard.

In conclusion, the present analysis suggests that both the web and paper versions of the MDHQ showed reasonable relative validity in terms of food intake against the 4-day weighed DR. The MDHQ had a satisfactory ability to estimate median intake and rank individuals according to consumption for many food groups, despite a limited ability to estimate food group intakes on an individual level. The MDHQ, which provides estimates of the dietary intake for each meal type (breakfast, lunch, dinner, and snacks), may be invaluable as a dietary assessment tool for future nutritional epidemiologic research on diet–disease relationships, with a particular focus on meal patterns and time of day of dietary intake, or chrono-nutrition research. The relative validity of MDHQ in other aspects of diet (e.g., nutrient level and diet quality) is currently examined to establish a more solid scientific basis.

## Figures and Tables

**Table 1:** Basic characteristics of the study population ^1^.

Variable	Women (*n* = 111)	Men (*n* = 111)
Age (years)	49.9 ± 10.7	51.7 ± 11.9
Body height (cm) ^2^	158.4 ± 5.4	170.2 ± 6.3
Body weight (kg) ^2^	56.9 ± 8.5	68.9 ± 11.9
Body mass index (kg/m^2^) ^3^	22.7 ± 3.3	23.8 ± 3.6
Education level (*n* (%))		
Junior high school or high school	28 (25.2)	41 (36.9)
College or technical school	55 (49.5)	22 (19.8)
University or higher	28 (25.2)	48 (43.2)
Current smoking status (*n* (%))		
Smoker	12 (10.8)	35 (31.5)
Nonsmoker	99 (89.2)	76 (68.4)
Total energy intake (kcal/day)		
4-day DR	1724 ± 335	2286 ± 493
Web version of MDHQ	1470 ± 349	1926 ± 517
Paper version of MDHQ	1509 ± 320	1895 ± 420

DR, dietary record; MDHQ, Meal-based Diet History Questionnaire. ^1^ Values are expressed as mean ± standard deviation, unless otherwise indicated. ^2^ Based on self-report. ^3^ Calculated using the self-reported body height and weight.

**Table 2:** Median estimates of daily intakes of Tier 1 food groups (in grams per day) derived from the 4-day weighed dietary record (DR) and those derived from the web version of the Meal-based Diet History Questionnaire (MDHQ) in 111 Japanese women, according to meal type ^1^.

Tier 1 Food Group	Breakfast		Lunch		Dinner		Snacks		Overall Diet	
		Web		Web		Web		Web		Web
	DR	MDHQ	DR	MDHQ	DR	MDHQ	DR	MDHQ	DR	MDHQ
Rice ^2^	25 (0, 78)	36 (0, 90)	76 (30, 115)	82 (57, 117)	88 (37, 131)	97 (39, 124)	0 (0, 0)	0 (0, 0) **	204 (133, 285)	218 (146, 274)
Bread ^2^	15 (0, 40)	18 (5, 44)	0 (0, 14)	5 (0, 10)	0 (0, 0)	0 (0, 0)	0 (0, 0)	0 (0, 0) **	30 (14, 51)	30 (15, 54)
Noodles ^2^	0 (0, 0)	0 (0, 0)	50 (0, 89)	25 (12, 50) ***	0 (0, 15)	20 (10, 21) ***	0 (0, 0)	0 (0, 0)	61 (23, 100)	46 (32, 78)
Miso soup ^2^	0 (0, 58)	12 (0, 77) **	0 (0, 27)	0 (0, 51) *	39 (0, 92)	52 (13, 103) **	0 (0, 0)	0 (0, 0)	98 (32, 155)	140 (52, 197) ***
Potatoes ^2^	0 (0, 2)	0 (0, 6) **	5 (0, 16)	5 (0, 11)	14 (3, 25)	15 (7, 22)	0 (0, 0)	0 (0, 0) ***	27 (15, 43)	24 (14, 34)
Pulses and nuts ^2^	4 (0, 25)	6 (0, 17)	3 (0, 11)	5 (0, 12)	20 (9, 35)	23 (15, 30)	0 (0, 1)	0 (0, 0) ***	44 (16, 87)	37 (24, 57) *
Vegetables ^2^	13 (0, 44)	12 (0, 60)	52 (26, 76)	38 (7, 66) ***	119 (85, 160)	121 (62, 140) ***	0 (0, 0)	0 (0, 0) ***	190 (143, 266)	167 (124, 224) ***
Pickled vegetables ^2^	0 (0, 0)	0 (0, 3) ***	0 (0, 2)	0 (0, 4) *	0 (0, 2)	3 (0, 10) ***	0 (0, 0)	0 (0, 0)	2 (0, 6)	7 (2, 19) ***
Fruit	6 (0, 53)	6 (0, 23) *	2 (0, 24)	0 (0, 20) **	1 (0, 18)	11 (0, 23) **	0 (0, 15)	6 (0, 24)	50 (15, 104)	47 (16, 95)
Fish and shellfish ^2^	0 (0, 6)	0 (0, 4) *	7 (1, 16)	3 (0, 12) ***	27 (13, 44)	19 (18, 28) ***	0 (0, 0)	0 (0, 0) **	45 (26, 63)	29 (19, 42) ***
Meat ^2^	3 (0, 10)	6 (0, 10)	21 (9, 33)	15 (5, 22) ***	51 (30, 69)	41 (25, 49) ***	0 (0, 0)	0 (0, 0) **	81 (58, 105)	58 (42, 75) ***
Eggs ^2^	7 (0, 23)	12 (3, 17)	10 (1, 18)	8 (2, 13) ***	7 (1, 14)	7 (3, 10) *	0 (0, 0)	0 (0, 0) ***	32 (19, 48)	26 (14, 37) ***
Dairy products	31 (0, 103)	54 (9, 123)	3 (0, 25)	6 (0, 24)	2 (0, 13)	6 (0, 12)	16 (0, 50)	5 (0, 34) ***	104 (38, 193)	106 (48, 155)
Confectioneries	0 (0, 2)	0 (0, 8)	0 (0, 13)	4 (0, 8)	0 (0, 0)	0 (0, 4)	11 (1, 28)	40 (14, 69) ***	25 (9, 50)	59 (29, 90) ***
Fruit and vegetable juice	0 (0, 0)	0 (0, 0)	0 (0, 0)	0 (0, 0)	0 (0, 0)	0 (0, 0) **	0 (0, 0)	0 (0, 0) *	0 (0, 0)	0 (0, 22) **
Alcoholic beverages ^3^	0 (0, 0)	0 (0, 0) ***	0 (0, 1)	0 (0, 0) ***	2 (0, 87)	0 (0, 55)	0 (0, 0)	0 (0, 0)	3 (0, 94)	4 (0, 63)
Soft drinks	0 (0, 0)	0 (0, 0) ***	0 (0, 0)	0 (0, 0)	0 (0, 0)	0 (0, 0)	0 (0, 52)	8 (0, 43)	0 (0, 65)	8 (0, 64)
Green tea	0 (0, 35)	0 (0, 80) ***	0 (0, 83)	28 (0, 88) *	0 (0, 50)	0 (0, 89)	0 (0, 131)	36 (0, 178) *	95 (0, 285)	172 (0, 433) **
Barley tea	0 (0, 10)	0 (0, 77) **	0 (0, 74)	29 (0, 144) ***	0 (0, 88)	30 (0, 179) **	0 (0, 157)	75 (0, 224) *	54 (0, 390)	235 (0, 562) ***
Oolong tea	0 (0, 0)	0 (0, 0) *	0 (0, 0)	0 (0, 0)	0 (0, 0)	0 (0, 0)	0 (0, 0)	0 (0, 0)	0 (0, 0)	0 (0, 0) **
Black tea	0 (0, 0)	0 (0, 0)	0 (0, 0)	0 (0, 0)	0 (0, 0)	0 (0, 0)	0 (0, 0)	0 (0, 28) **	0 (0, 47)	0 (0, 53)
Coffee	45 (0, 155)	149 (15, 205) ***	0 (0, 40)	27 (0, 102) ***	0 (0, 0)	0 (0, 0) ***	91 (0, 181)	103 (38, 213) **	206 (86, 362)	312 (173, 460) ***
Water	0 (0, 25)	26 (0, 175) ***	0 (0, 0)	0 (0, 146) ***	0 (0, 0)	0 (0, 148) ***	50 (0, 159)	146 (0, 357) **	88 (0, 272)	255 (51, 729) ***
Breakfast cereals ^4^	0 (0, 0)	0 (0, 0) *	0 (0, 0)	0 (0, 0)	0 (0, 0)	0 (0, 0)	0 (0, 0)	0 (0, 0)	0 (0, 0)	0 (0, 0)

^1^ Values are expressed medians (25th and 75th percentiles). The values derived from the MDHQ were compared with those derived from the DR using the Wilcoxon signed-rank test: * *p* < 0.05, ** *p* < 0.01, and *** *p* < 0.001. ^2^ Not assessed for snacks in the MDHQ; the intake for snacks was 0 g/day for all participants. ^3^ Not assessed for breakfast in the MDHQ; the breakfast intake was 0 g/day in all participants. ^4^ Only assessed for breakfast in the MDHQ; the intake for all other eating occasions was 0 g/day in all participants.

**Table 3:** Median estimates of daily intakes of Tier 1 food groups (in grams per day) derived from the 4-day weighed dietary record (DR) and those derived from the web version of the Meal-based Diet History Questionnaire (MDHQ) in 111 Japanese men, according to meal type ^1^.

Tier 1 Food Group	Breakfast		Lunch		Dinner		Snacks		Overall Diet	
		Web		Web		Web		Web		Web
	DR	MDHQ	DR	MDHQ	DR	MDHQ	DR	MDHQ	DR	MDHQ
Rice ^2^	38 (0, 119)	44 (0, 118)	125 (63, 174)	139 (82, 183)	137 (78, 198)	139 (83, 178)	0 (0, 0)	0 (0, 0) *	313 (232, 389)	315 (250, 384)
Bread ^2^	15 (0, 39)	21 (0, 47) *	0 (0, 11)	0 (0, 13)	0 (0, 0)	0 (0, 0)	0 (0, 0)	0 (0, 0) ***	31 (14, 55)	34 (10, 69)
Noodles ^2^	0 (0, 0)	0 (0, 0)	63 (2, 135)	27 (13, 53) ***	0 (0, 30)	23 (12, 48) **	0 (0, 0)	0 (0, 0)	91 (46, 170)	71 (36, 101) ***
Miso soup ^2^	0 (0, 69)	0 (0, 77)	0 (0, 0)	0 (0, 46) *	36 (0, 107)	51 (0, 103)	0 (0, 0)	0 (0, 0)	101 (39, 174)	129 (51, 187)
Potatoes ^2^	0 (0, 4)	0 (0, 3)	4 (0, 14)	3 (0, 9) ***	15 (3, 29)	17 (8, 24)	0 (0, 0)	0 (0, 0) ***	28 (15, 50)	22 (10, 37) ***
Pulses and nuts ^2^	5 (0, 20)	3 (0, 12) **	0 (0, 6)	3 (0, 11)	23 (9, 50)	18 (9, 27) ***	0 (0, 0)	0 (0, 0) ***	39 (20, 75)	30 (17, 50) ***
Vegetables ^2^	13 (0, 46)	6 (0, 49)	52 (25, 78)	37 (0, 66) ***	145 (110, 185)	127 (69, 157) ***	0 (0, 0)	0 (0, 0) ***	216 (165, 295)	173 (106, 241) ***
Pickled vegetables ^2^	0 (0, 0)	0 (0, 2) **	0 (0, 4)	0 (0, 7)	0 (0, 3)	2 (0, 9) ***	0 (0, 0)	0 (0, 0)	3 (0, 12)	9 (2, 19) ***
Fruit	0 (0, 30)	0 (0, 22) ***	0 (0, 3)	0 (0, 9)	0 (0, 15)	6 (0, 22)	0 (0, 0)	0 (0, 14) *	34 (1, 74)	24 (0, 79)
Fish and shellfish ^2^	0 (0, 6)	0 (0, 5) **	8 (1, 20)	7 (0, 20)	33 (20, 64)	26 (23, 37) ***	0 (0, 0)	0 (0, 0) **	55 (33, 83)	39 (27, 53) ***
Meat ^2^	7 (0, 15)	4 (0, 12)	27 (14, 49)	21 (13, 32) ***	72 (45, 94)	41 (32, 53) ***	0 (0, 0)	0 (0, 0) **	112 (82, 145)	72 (54, 87) ***
Eggs ^2^	13 (0, 26)	6 (0, 19) **	12 (2, 24)	9 (2, 16) ***	8 (1, 16)	4 (2, 9) **	0 (0, 0)	0 (0, 0) ***	44 (27, 56)	28 (16, 42) ***
Dairy products	8 (0, 77)	9 (0, 114)	1 (0, 6)	0 (0, 12)	2 (0, 15)	0 (0, 10)	2 (0, 26)	0 (0, 14) **	57 (13, 169)	51 (5, 131) ***
Confectioneries	0 (0, 13)	0 (0, 4) *	0 (0, 6)	0 (0, 9)	0 (0, 0)	0 (0, 0)	6 (0, 24)	28 (7, 59) ***	24 (7, 48)	49 (16, 80) ***
Fruit and vegetable juice	0 (0, 0)	0 (0, 0) **	0 (0, 0)	0 (0, 0)	0 (0, 0)	0 (0, 0) *	0 (0, 0)	0 (0, 0)	0 (0, 0)	0 (0, 18) ***
Alcoholic beverages ^3^	0 (0, 0)	0 (0, 0) ***	0 (0, 1)	0 (0, 0) *	5 (0, 253)	60 (0, 284) *	0 (0, 0)	0 (0, 54)	69 (2, 398)	140 (0, 410) **
Soft drinks	0 (0, 0)	0 (0, 0) *	0 (0, 0)	0 (0, 0)	0 (0, 0)	0 (0, 0)	0 (0, 85)	0 (0, 73)	0 (0, 125)	0 (0, 107)
Green tea	0 (0, 0)	0 (0, 86) ***	0 (0, 70)	0 (0, 151) *	0 (0, 0)	0 (0, 31)	0 (0, 125)	0 (0, 167)	85 (0, 277)	142 (0, 412) *
Barley tea	0 (0, 0)	0 (0, 49) *	0 (0, 94)	0 (0, 135)	0 (0, 70)	0 (0, 127) *	0 (0, 110)	15 (0, 213)	45 (0, 431)	86 (0, 481)
Oolong tea	0 (0, 0)	0 (0, 0) **	0 (0, 0)	0 (0, 0)	0 (0, 0)	0 (0, 0)	0 (0, 0)	0 (0, 0)	0 (0, 0)	0 (0, 0)
Black tea	0 (0, 0)	0 (0, 0) *	0 (0, 0)	0 (0, 0) *	0 (0, 0)	0 (0, 0)	0 (0, 0)	0 (0, 0)	0 (0, 0)	0 (0, 0)
Coffee	42 (0, 155)	122 (0, 210) ***	0 (0, 41)	0 (0, 91) ***	0 (0, 0)	0 (0, 0) *	76 (0, 250)	137 (0, 325)	215 (67, 395)	277 (153, 581) ***
Water	0 (0, 5)	0 (0, 181) ***	0 (0, 0)	33 (0, 214) ***	0 (0, 0)	30 (0, 209) ***	15 (0, 162)	123 (0, 372) **	61 (0, 251)	319 (14, 791) ***
Breakfast cereals ^4^	0 (0, 0)	0 (0, 0)	0 (0, 0)	0 (0, 0)	0 (0, 0)	0 (0, 0)	0 (0, 0)	0 (0, 0)	0 (0, 0)	0 (0, 0)

^1^ Values are expressed as medians (25th and 75th percentiles). The values derived from the MDHQ were compared with those derived from the DR using the Wilcoxon signed-rank test: * *p* < 0.05, ** *p* < 0.01, and *** *p* < 0.001. ^2^ Not assessed for snacks in the MDHQ; the intake of snacks was 0 g/day in all participants. ^3^ Not assessed for breakfast in the MDHQ; the breakfast intake was 0 g/day in all participants. ^4^ Only assessed for breakfast in the MDHQ; the intake in all other eating occasions was 0 g/day in all participants.

**Table 4:** Spearman’s correlation coefficients between estimates of daily intakes of Tier 1 food groups (in grams per day) derived from the 4-day weighed dietary record and those derived from the web version of the Meal-based Diet History Questionnaire (MDHQ) in 111 Japanese women and 111 Japanese men, according to meal type ^1^.

Tier 1 Food Group	Women					Men				
	Breakfast	Lunch	Dinner	Snacks	Overall Diet	Breakfast	Lunch	Dinner	Snacks	Overall Diet
Rice ^2^	0.77 ***	0.43 ***	0.51 ***	−−−	0.61 ***	0.75 ***	0.56 ***	0.59 ***	−−−	0.54 ***
Bread ^2^	0.49 ***	0.18	0.26 **	−−−	0.41 ***	0.68 ***	0.39 ***	0.15	−−−	0.56 ***
Noodles ^2^	0.19 *	0.42 ***	0.17	−−−	0.34 ***	0.45 ***	0.46 ***	0.07	−−−	0.39 ***
Miso soup ^2^	0.77 ***	0.48 ***	0.65 ***	−−−	0.65 ***	0.74 ***	0.34 ***	0.68 ***	−−−	0.67 ***
Potatoes ^2^	0.51 ***	0.10	0.23 *	−−−	0.21 *	0.47 ***	0.23 *	0.18	−−−	0.25 **
Pulses and nuts ^2^	0.64 ***	0.35 ***	0.27 **	−−−	0.46 ***	0.70 ***	0.07	0.14	−−−	0.39 ***
Vegetables ^2^	0.63 ***	0.34 ***	0.26 **	−−−	0.37 ***	0.60 ***	0.35 ***	0.31 **	−−−	0.46 ***
Pickled vegetables ^2^	0.45 ***	0.25 **	0.07	−−−	0.43 ***	0.42 ***	0.26 **	0.11	−−−	0.29 **
Fruit	0.57 ***	0.44 ***	0.38 ***	0.27 **	0.55 ***	0.73 ***	0.41 ***	0.21 *	0.36 ***	0.64 ***
Fish and shellfish ^2^	0.61 ***	0.25 **	0.28 **	−−−	0.41 ***	0.53 ***	0.19	0.25 **	−−−	0.30 **
Meat ^2^	0.54 ***	0.29 **	0.16	−−−	0.31 ***	0.61 ***	0.41 ***	0.15	−−−	0.21 *
Eggs ^2^	0.47 ***	0.29 **	0.37 ***	−−−	0.49 ***	0.65 ***	0.29 **	0.24 *	−−−	0.66 ***
Dairy products	0.62 ***	0.19 *	0.24 *	0.39 ***	0.61 ***	0.61 ***	0.27 **	0.13	0.22 *	0.65 ***
Confectioneries	0.31 **	0.22 *	0.17	0.50 ***	0.43 ***	0.33 ***	0.50 ***	0.04	0.34 ***	0.27 **
Fruit and vegetable juice	0.18	0.21 *	0.39 ***	0.26 **	0.45 ***	0.60 ***	0.26 **	0.25 **	0.19	0.55 ***
Alcoholic beverages ^3^	−−−	−0.13	0.63 ***	0.45 ***	0.73 ***	−−−	0.05	0.72 ***	0.50 ***	0.82 ***
Soft drinks	0.29 **	0.39 ***	0.05	0.48 ***	0.48 ***	0.03	0.28 **	0.28 **	0.40 ***	0.36 ***
Green tea	0.55 ***	0.51 ***	0.51 ***	0.37 ***	0.53 ***	0.67 ***	0.33 ***	0.33 ***	0.41 ***	0.55 ***
Barley tea	0.55 ***	0.59 ***	0.58 ***	0.59 ***	0.64 ***	0.50 ***	0.46 ***	0.56 ***	0.43 ***	0.57 ***
Oolong tea	−0.03	0.38 ***	0.50 ***	0.29 **	0.43 ***	0.42 ***	0.36 ***	0.43 ***	0.29 **	0.33 ***
Black tea	0.45 ***	0.16	0.39 ***	0.53 ***	0.47 ***	0.59 ***	0.39 ***	0.18	0.38 ***	0.45 ***
Coffee	0.70 ***	0.46 ***	0.47 ***	0.75 ***	0.78 ***	0.72 ***	0.59 ***	0.43 ***	0.71 ***	0.77 ***
Water	0.29 **	0.30 **	0.13	0.54 ***	0.59 ***	0.13	0.16	0.21 *	0.52 ***	0.53 ***
Breakfast cereals ^4^	0.54 ***	−−−	−−−	−−−	0.51 ***	0.54 ***	−−−	−−−	−−−	0.46 ***

^1^ Values are expressed as Spearman’s correlation coefficients. * *p* < 0.05, ** *p* < 0.01, and *** *p* < 0.001. ^2^ Not assessed for snacks in the MDHQ; the intake of snacks was 0 g/day in all participants. ^3^ Not assessed for breakfast in the MDHQ; the breakfast intake was 0 g/day in all participants. ^4^ Only assessed for breakfast in the MDHQ; the intake in all other eating occasions was 0 g/day in all participants.

**Table 5:** Median estimates of overall intakes of Tier 2 food groups (in grams per day) derived from the 4-day weighed dietary record (DR) and those derived from the web version of the Meal-based Diet History Questionnaire (MDHQ) and Spearman’s correlation coefficients between estimates derived from the DR and the web version of MDHQ in 111 Japanese women and 111 Japanese men ^1^.

Tier 2 Food Group	Women			Men		
	DR	Web MDHQ	Correlation ^2^	DR	Web MDHQ	Correlation ^2^
Rice						
White rice	203 (131, 285)	208 (135, 267)	0.60 ***	311 (231, 389)	301 (220, 355) *	0.53 ***
Brown rice	0 (0, 0)	0 (0, 9) ***	0.24 *	0 (0, 0)	0 (0, 13) ***	0.27 **
Bread						
White bread	30 (12, 49)	24 (9, 42) *	0.39 ***	30 (13, 55)	27 (7, 60)	0.54 ***
Wholegrain bread	0 (0, 0)	2 (0, 9) ***	0.19 *	0 (0, 0)	0 (0, 7) ***	0.07
Noodles						
Wheat noodles	0 (0, 23)	30 (15, 46) ***	0.16	0 (0, 45)	32 (12, 55) **	0.17
Chinese noodles	0 (0, 36)	5 (0, 15)	0.23 *	0 (0, 52)	12 (2, 29)	0.24 *
Instant noodles	0 (0, 0)	0 (0, 3)	0.24 **	0 (0, 54)	1 (0, 5) *	0.33 ***
Spaghetti	0 (0, 25)	4 (1, 10)	0.24 *	0 (0, 22)	2 (0, 9)	0.21 *
Buckwheat noodles	0 (0, 0)	1 (0, 5) ***	0.24 *	0 (0, 0)	1 (0, 6) ***	0.25 **
Pulses and nuts						
Soy milk	0 (0, 0)	0 (0, 0)	0.53 ***	0 (0, 0)	0 (0, 0)	0.35 ***
Tofu (i.e., soybean curd)	18 (3, 41)	29 (17, 45) **	0.25 **	21 (3, 46)	23 (11, 36)	0.27 **
Natto (i.e., fermented soybeans)	0 (0, 13)	3 (1, 8) *	0.54 ***	0 (0, 10)	2 (0, 6)	0.63 ***
Tofu products	1 (0, 4)	3 (1, 6)	0.36 ***	2 (0, 6)	1 (0, 4) *	0.26 **
Peanuts and nuts	1 (0, 6)	0 (0, 0) ***	0.16	1 (0, 6)	0 (0, 0) ***	0.32 ***
All other pulses and nuts	0 (0, 2)	0 (0, 1)	0.21 *	0 (0, 1)	0 (0, 1)	0.09
Vegetables						
Edamame (i.e., immature soybeans) and peas	0 (0, 2)	1 (0, 2)	0.13	0 (0, 3)	0 (0, 2)	0.30 **
Seaweeds	4 (1, 11)	5 (2, 9)	0.16	4 (1, 9)	4 (1, 8)	0.27 **
Pumpkins	0 (0, 10)	3 (0, 5)	0.36 ***	0 (0, 13)	2 (0, 5) *	0.46 ***
Mushrooms	10 (2, 18)	7 (4, 14) **	0.29 **	10 (2, 21)	6 (1, 11) ***	0.28 **
Cabbage	19 (9, 40)	14 (8, 25) **	0.22 *	29 (11, 45)	16 (8, 32) ***	0.36 ***
Cucumbers	10 (0, 22)	11 (6, 17)	0.18	10 (0, 20)	9 (4, 17)	0.32 ***
Bitter melon	0 (0, 0)	0 (0, 1)	0.36 ***	0 (0, 0)	0 (0, 1)	0.47 ***
Burdock	0 (0, 4)	0 (0, 1) **	0.32 ***	0 (0, 5)	0 (0, 1) ***	0.06
Radishes	6 (0, 18)	7 (1, 11)	0.37 ***	8 (0, 15)	7 (1, 15)	0.21 *
Onions	22 (13, 35)	28 (12, 39)	0.25 **	28 (13, 41)	24 (12, 40)	0.29 **
Chinese cabbage	0 (0, 0)	0 (0, 2)	0.24 **	0 (0, 0)	0 (0, 2)	0.13
Tomatoes	8 (0, 24)	24 (11, 37) ***	0.37 ***	9 (0, 20)	18 (6, 40) ***	0.33 ***
Eggplants	0 (0, 11)	8 (4, 13)	0.40 ***	0 (0, 13)	6 (2, 12)	0.33 ***
Carrots	10 (4, 16)	13 (6, 21) *	0.38 ***	10 (4, 18)	12 (5, 24)	0.33 ***
Green peppers	2 (0, 8)	3 (2, 6)	0.26 **	3 (0, 10)	3 (1, 4) **	0.30 **
Broccoli	0 (0, 4)	2 (0, 3)	0.36 ***	0 (0, 6)	1 (0, 4)	0.35 ***
Green leafy vegetables	10 (3, 19)	14 (7, 24) *	0.32 ***	10 (3, 20)	10 (2, 18)	0.28 **
Bean sprouts	6 (0, 14)	3 (1, 6) ***	0.35 ***	5 (0, 21)	2 (1, 5) ***	0.36 ***
Lettuce	3 (0, 10)	4 (1, 8)	0.30 **	4 (0, 10)	4 (1, 7)	0.38 ***
All other vegetables	12 (4, 30)	1 (1, 1) ***	0.16	17 (6, 28)	1 (1, 2) ***	−0.11
Fruit						
Strawberries	0 (0, 0)	0 (0, 0) ***	0.05	0 (0, 0)	0 (0, 0) **	−0.15
Persimmons	0 (0, 0)	0 (0, 0)	−0.05	0 (0, 0)	0 (0, 0)	0.15
Citrus	0 (0, 3)	2 (0, 9) **	−0.16	0 (0, 1)	0 (0, 6) **	0.16
Kiwi fruit	0 (0, 0)	0 (0, 1)	0.40 ***	0 (0, 0)	0 (0, 0)	0.40 ***
Watermelon	0 (0, 0)	0 (0, 1) ***	0.12	0 (0, 0)	0 (0, 0) ***	0.16
Pears	0 (0, 15)	1 (0, 10)	0.25 **	0 (0, 10)	0 (0, 5) *	0.35 ***
Bananas	0 (0, 19)	5 (0, 27) *	0.58 ***	0 (0, 18)	1 (0, 16)	0.64 ***
Grapes	0 (0, 20)	2 (0, 8) *	0.16	0 (0, 13)	0 (0, 4) *	0.28 **
Melon	0 (0, 0)	0 (0, 1) ***	0.20 *	0 (0, 0)	0 (0, 0) ***	0.15
Peaches	0 (0, 0)	0 (0, 3) ***	0.11	0 (0, 0)	0 (0, 1) **	0.09
Apples	0 (0, 1)	0 (0, 5)	0.42 ***	0 (0, 0)	0 (0, 4) **	0.32 ***
All other fruit	0 (0, 6)	1 (0, 1)	−0.01	0 (0, 4)	1 (0, 1)	0.08
Fish and shellfish						
Oily fish	0 (0, 19)	6 (1, 9)	0.09	5 (0, 24)	8 (2, 14)	0.20 *
Red meat fish	0 (0, 7)	4 (1, 7) *	0.17	0 (0, 5)	6 (1, 10) **	0.17
Squid and octopus	0 (0, 4)	0 (0, 2)	0.22 *	0 (0, 5)	1 (0, 4)	0.41 ***
Eel	0 (0, 0)	0 (0, 0) ***	0.01	0 (0, 0)	0 (0, 0) ***	−0.09
Shrimp and crab	0 (0, 3)	0 (0, 2)	0.03	0 (0, 5)	1 (0, 3)	0.15
Shellfish	0 (0, 0)	0 (0, 1) *	0.16	0 (0, 0)	0 (0, 1)	0.25 **
Small fish with bones	0 (0, 0)	0 (0, 0) *	0.32 ***	0 (0, 0)	0 (0, 0) *	0.23 *
Fish eggs	0 (0, 0)	0 (0, 0)	0.28 **	0 (0, 0)	0 (0, 0)	0.05
Dried fish	0 (0, 1)	1 (0, 3)	0.15	0 (0, 0)	1 (0, 4)	0.21 *
Salmon	1 (0, 14)	3 (1, 5) *	0.25 **	3 (0, 16)	4 (1, 8)	0.04
White meat fish	0 (0, 5)	1 (1, 6)	0.11	0 (0, 12)	2 (1, 9)	0.19 *
Ground fish meat products	0 (0, 7)	4 (1, 6)	0.23 *	3 (0, 10)	4 (1, 7)	0.12
Canned tuna	0 (0, 3)	0 (0, 1)	0.35 ***	0 (0, 3)	0 (0, 1)	0.31 **
All other fish and shellfish	0 (0, 0)	0 (0, 0) ***	0.00	0 (0, 0)	0 (0, 0) ***	−0.18
Meat						
Beef	6 (0, 18)	7 (1, 11) *	0.31 ***	10 (1, 24)	9 (2, 15) *	0.24 *
Chicken	25 (11, 42)	13 (9, 23) ***	0.24 *	36 (18, 71)	17 (12, 26) ***	0.13
Processed meat	8 (2, 15)	8 (4, 15)	0.42 ***	11 (3, 19)	9 (5, 20)	0.38 ***
Pork	28 (12, 42)	26 (15, 33)	0.21 *	34 (20, 54)	25 (18, 38) **	0.04
Liver	0 (0, 0)	0 (0, 0) ***	0.17	0 (0, 0)	0 (0, 0) ***	0.10
All other meat	0 (0, 0)	0 (0, 0) ***	0.12	0 (0, 0)	0 (0, 0) ***	−0.10
Dairy products						
Ice cream	0 (0, 8)	1 (0, 5)	0.38 ***	0 (0, 0)	0 (0, 4)	0.26 **
Cheese	3 (0, 8)	2 (0, 4)	0.20 *	2 (0, 8)	0 (0, 3) **	0.11
Low-fat milk	0 (0, 0)	0 (0, 0)	0.49 ***	0 (0, 0)	0 (0, 0)	0.64 ***
Yogurt	29 (0, 103)	16 (1, 51) ***	0.59 ***	8 (0, 64)	1 (0, 25) ***	0.60 ***
Full-fat milk	17 (0, 62)	38 (3, 88) ***	0.58 ***	0 (0, 27)	1 (0, 32) **	0.58 ***
All other dairy products	0 (0, 3)	0 (0, 1)	0.06	0 (0, 3)	0 (0, 1) *	0.01
Confectioneries						
Candies, caramels, and chewing gum	0 (0, 0)	0 (0, 0) ***	0.29 **	0 (0, 0)	0 (0, 1) ***	0.19 *
Japanese bread with a sweet filling	0 (0, 0)	3 (0, 8) ***	0.17	0 (0, 10)	2 (0, 8)	0.34 ***
Snacks made from wheat flour	0 (0, 0)	2 (0, 5) ***	0.37 ***	0 (0, 0)	1 (0, 6) ***	0.29 **
Jellies	0 (0, 0)	0 (0, 1) **	0.11	0 (0, 0)	0 (0, 0) ***	0.18
Rice crackers	0 (0, 2)	5 (1, 10) ***	0.47 ***	0 (0, 0)	4 (0, 10) ***	0.37 ***
Chocolates	1 (0, 5)	7 (1, 17) ***	0.33 ***	0 (0, 3)	3 (0, 13) ***	0.22 *
Biscuits and cookies	0 (0, 2)	2 (0, 6) ***	0.24 *	0 (0, 0)	0 (0, 3) ***	0.20 *
Cakes	0 (0, 13)	11 (3, 25) ***	0.26 **	0 (0, 6)	7 (1, 18) ***	0.29 **
Japanese sweets	0 (0, 13)	4 (1, 16) *	0.17	0 (0, 10)	2 (0, 12) *	0.28 **
Alcoholic beverages						
Beer	0 (0, 0)	0 (0, 26)	0.77 ***	0 (0, 177)	48 (0, 304) **	0.78 ***
Sake	0 (0, 0)	0 (0, 0)	0.64 ***	0 (0, 0)	0 (0, 0)	0.30 **
Shochu (i.e., Japanese distilled beverages)	0 (0, 0)	0 (0, 2)	0.77 ***	0 (0, 80)	0 (0, 100)	0.63 ***
Wine	0 (0, 0)	0 (0, 0)	0.25 **	0 (0, 0)	0 (0, 0)	0.60 ***
Whiskey and other spirits	0 (0, 0)	0 (0, 0)	--- ^3^	0 (0, 0)	0 (0, 0)	0.70 ***

^1^ Values are expressed as medians (25th and 75th percentiles) unless otherwise indicated. The values derived from the MDHQ were compared with those derived from the DR using the Wilcoxon signed-rank test: * *p* < 0.05, ** *p* < 0.01, *** *p* < 0.001. ^2^ Spearman’s correlation coefficients: * *p* < 0.05, ** *p* < 0.01, *** *p* < 0.001. ^3^ Not available because the intake derived from the DR was 0 g/day in all participants.

## Data Availability

The datasets generated and analyzed during the present study are not publicly available because of privacy and ethical restrictions imposed by the Ethics Committee of the University of Tokyo, Faculty of Medicine, but are available from the corresponding author upon reasonable request. The web and paper versions of the MDHQ used in this study are available from the corresponding author upon request.

## Supplementary Materials

The following supporting information can be downloaded at: https://www.mdpi.com/article/10.3390/nu14153193/s1, Table S1: List of food groups estimated from the Meal-based Diet History Questionnaire; Table S2: Mean estimates of daily intakes of Tier 1 food groups (in grams per day) derived from the 4-day weighed dietary record (DR) and those derived from the web version of the Meal-based Diet History Questionnaire (MDHQ) in 111 Japanese women, according to meal type; Table S3: Mean estimates of daily intakes of Tier 1 food groups (in grams per day) derived from the 4-day weighed dietary record (DR) and those derived from the web version of the Meal-based Diet History Questionnaire (MDHQ) in 111 Japanese men, according to meal type; Table S4: Bland–Altman analysis for estimates of daily intakes of Tier 1 food groups (in grams per day) derived from the 4-day weighed dietary record and those derived from the web version of the Meal-based Diet History Questionnaire (MDHQ) in 111 Japanese women, according to meal type; Table S5: Bland–Altman analysis for estimates of daily intakes of Tier 1 food groups (in grams per day) derived from the 4-day weighed dietary record (DR) and those derived from the web version of the Meal-based Diet History Questionnaire (MDHQ) in 111 Japanese men, according to meal type; Table S6: Mean estimates of overall intakes of Tier 2 food groups (in grams per day) derived from the 4-day weighed dietary record (DR) and those derived from the web version of the Meal-based Diet History Questionnaire (MDHQ) and Bland–Altman analysis for these estimates in 111 Japanese women and 111 Japanese men; Table S7: Median estimates of daily intakes of Tier 1 food groups (in grams per day) derived from the paper version of the Meal-based Diet History Questionnaire (MDHQ) in 111 Japanese women and 111 Japanese men, according to meal type; Table S8: Mean estimates of daily intakes of Tier 1 food groups (in grams per day) derived from the paper version of the Meal-based Diet History Questionnaire (MDHQ) in 111 Japanese women and 111 Japanese men, according to meal type; Table S9: Spearman correlation coefficients between estimates of daily intakes of Tier 1 food groups (in grams per day) derived from the 4-day weighed dietary record and those derived from the paper version of the Meal-based Diet History Questionnaire (MDHQ) in 111 Japanese women and 111 Japanese men, according to meal type; Table S10: Bland–Altman analysis for estimates of daily intakes of Tier 1 food groups (in grams per day) derived from the 4-day weighed dietary record (DR) and those derived from the paper version of the Meal-based Diet History Questionnaire (MDHQ) in 111 Japanese women, according to meal type; Table S11: Bland–Altman analysis for estimates of daily intakes of Tier 1 food groups (in grams per day) derived from the 4-day weighed dietary record (DR) and those derived from the paper version of the Meal-based Diet History Questionnaire (MDHQ) in 111 Japanese men, according to meal type; Table S12: Estimates of overall intakes of Tier 2 food groups (in grams per day) derived from the 4-day weighed dietary record (DR) and those derived from the paper version of the Meal-based Diet History Questionnaire (MDHQ), Spearman correlation coefficients, and Bland–Altman analysis in 111 Japanese women and 111 Japanese men.
