# Supplementary material for: Relative Validity of Food Intake in Each Meal Type and Overall Food Intake Derived Using the Meal-Based Diet History Questionnaire against the 4-Day Weighed Dietary Record in Japanese Adults
Source: Nutrients. 2022 Aug 4;14(15):3193. doi: 10.3390/nu14153193 (PMC9370650; doi:10.3390/nu14153193)
Supplement: Supplementary file 1 [file nutrients-14-03193-s001.zip › nutrients-1831694-SI.pdf]

Table S1. List of food groups estimated from the Meal-based Diet History Questionnaire.

Tier 1 food group	Tier 2 food group
Rice	White rice; brown rice
Bread	White bread; wholegrain bread
Noodles	Wheat noodles; Chinese noodles; instant noodles; spaghetti; buckwheat noodles
Miso soup	---
Potatoes	---
Pulses and nuts	Soy milk; tofu (i.e., soybean curd); natto (i.e., fermented soybeans); tofu products; peanuts and nuts; all other pulses and nuts
Vegetables	Edamame (i.e., immature soybeans) and peas; seaweeds; pumpkins; mushrooms; cabbage; cucumbers; bitter melon; burdock; radishes; onions; Chinese cabbage; tomatoes; eggplants; carrots; green peppers; broccoli; green leafy vegetables; bean sprouts; lettuce; all other vegetables
Pickled vegetables	---
Fruits	Strawberries; persimmons; citrus; kiwi fruit; watermelon; pears; bananas; grapes; melon; peaches; apples; all other fruit
Fish and shellfish	Oily fish; red meat fish; squid and octopus; eel; shrimp and crab; shellfish; small fish with bones; fish eggs; dried fish; salmon; white meat fish; ground fish meat products; canned tuna; all other fish and shellfish
Meat	Liver; processed meat; beef; chicken; pork; all other meat
Eggs	---
Dairy products	Ice cream; cheese; low-fat milk; yogurt; full-fat milk; all other dairy products
Confectioneries	Candies, caramels, and chewing gum; Japanese bread with a sweet filling; snacks made from wheat flour; jellies; rice crackers; chocolates; biscuits and cookies; cakes; Japanese sweets
Fruit and vegetable juice	---
Alcoholic beverages	Beer; sake; shochu (i.e., Japanese distilled beverages); wine; whiskey and other spirits
Soft drinks	---
Green tea	---
Barley tea	---
Oolong tea	---
Black tea	---
Coffee	---
Water	---
Breakfast cereals	---
Seasonings [1]	Sugar added to coffee and tea; salt for cooking; vegetable oils; sugar for cooking; salt for soup; soy sauce; jam for bread; fat spread for bread; mayonnaise and dressing

[1] Not assessed in this study.

Table S2. Mean estimates of daily intakes of Tier 1 food groups (in grams per day) derived from the 4-day weighed dietary record (DR) and those derived from the web version of the Meal-based Diet History Questionnaire (MDHQ) in 111 Japanese women, according to meal type [1].

	Breakfast		Lunch		Dinner		Snacks		Overall diet	
**Tier 1 food group**	**DR**	**Web MDHQ**	**DR**	**Web MDHQ**	**DR**	**Web MDHQ**	**DR**	**Web MDHQ**	**DR**	**Web MDHQ**
Rice [2]	45 ± 61	47 ± 46	76 ± 51	78 ± 43	86 ± 57	85 ± 44	1 ± 4	0 ± 0*	208 ± 110	210 ± 85
Bread [2]	24 ± 26	25 ± 22	9 ± 17	8 ± 11	1 ± 5	2 ± 6	1 ± 4	0 ± 0**	35 ± 30	35 ± 27
Noodles [2]	2 ± 12	1 ± 3	54 ± 56	35 ± 32***	13 ± 24	20 ± 17**	0 ± 1	0 ± 0	69 ± 60	55 ± 36*
Miso soup [2]	35 ± 58	46 ± 59**	17 ± 35	28 ± 43**	55 ± 57	68 ± 58**	0 ± 4	0 ± 0	108 ± 86	141 ± 100***
Potatoes [2]	3 ± 5	5 ± 7**	10 ± 14	7 ± 7**	17 ± 17	15 ± 9	1 ± 4	0 ± 0*	32 ± 24	26 ± 17*
Pulses and nuts [2]	17 ± 30	12 ± 13	11 ± 21	7 ± 7	26 ± 27	23 ± 14	5 ± 16	0 ± 0**	59 ± 55	42 ± 24***
Vegetables [2]	28 ± 39	30 ± 32	59 ± 44	41 ± 32***	124 ± 59	99 ± 45***	2 ± 8	0 ± 0**	213 ± 108	169 ± 75***
Pickled vegetables [2]	1 ± 4	3 ± 5***	2 ± 4	3 ± 5	3 ± 9	6 ± 7**	0 ± 0	0 ± 0	6 ± 12	12 ± 13***
Fruit	27 ± 36	19 ± 25**	15 ± 25	10 ± 14**	12 ± 20	16 ± 18*	14 ± 28	15 ± 24	67 ± 66	60 ± 49
Fish and shellfish [2]	5 ± 11	3 ± 5**	12 ± 18	6 ± 7***	30 ± 22	22 ± 11***	0 ± 3	0 ± 0	48 ± 32	31 ± 17***
Meat [2]	7 ± 10	7 ± 7	25 ± 19	15 ± 11***	52 ± 30	37 ± 15***	0 ± 2	0 ± 0*	84 ± 39	59 ± 24***
Eggs [2]	13 ± 16	12 ± 11	12 ± 12	8 ± 7***	10 ± 10	7 ± 5**	1 ± 2	0 ± 0**	35 ± 22	27 ± 15***
Dairy products	64 ± 77	62 ± 53	21 ± 42	15 ± 22	9 ± 15	12 ± 18	37 ± 54	21 ± 31***	130 ± 115	111 ± 80*
Confectioneries	6 ± 14	6 ± 11	9 ± 15	7 ± 12	2 ± 6	4 ± 9	17 ± 18	45 ± 34***	34 ± 30	62 ± 44***
Fruit and vegetable juice	4 ± 20	8 ± 29	5 ± 22	5 ± 16	2 ± 9	4 ± 17**	6 ± 25	12 ± 39	17 ± 41	29 ± 59*
Alcoholic beverages [3]	0 ± 0	0 ± 0**	1 ± 1	2 ± 12	73 ± 169	67 ± 151	18 ± 69	21 ± 67	92 ± 201	89 ± 188
Soft drinks	1 ± 8	6 ± 17*	6 ± 22	7 ± 20	3 ± 16	5 ± 18	33 ± 55	33 ± 58	43 ± 67	51 ± 95
Green tea	24 ± 55	48 ± 70***	42 ± 56	57 ± 71*	40 ± 73	52 ± 78	81 ± 155	109 ± 143	186 ± 253	267 ± 303**
Barley tea	28 ± 70	44 ± 65*	46 ± 75	68 ± 79**	55 ± 95	79 ± 86**	115 ± 208	140 ± 168	244 ± 375	331 ± 355**
Oolong tea	0 ± 2	5 ± 23*	5 ± 18	8 ± 32	6 ± 35	11 ± 39	12 ± 52	24 ± 85	23 ± 83	48 ± 150*
Black tea	13 ± 42	14 ± 39	9 ± 25	9 ± 27	3 ± 16	8 ± 30	25 ± 66	40 ± 86*	50 ± 102	71 ± 155*
Coffee	85 ± 95	118 ± 90***	29 ± 52	55 ± 65***	7 ± 21	22 ± 49***	116 ± 129	152 ± 144***	236 ± 202	346 ± 262***
Water	18 ± 36	73 ± 79***	10 ± 23	67 ± 84***	8 ± 21	61 ± 83***	156 ± 275	200 ± 197	191 ± 294	400 ± 390***
Breakfast cereals [4]	1 ± 4	3 ± 9*	0 ± 2	0 ± 0	0 ± 3	0 ± 0	0 ± 0	0 ± 0	2 ± 5	3 ± 9

[1] Values are means ± standard deviations. The values derived from the MDHQ were compared with those derived from the DR using the paired *t*-test: * p <0.05, ** p <0.01, *** p <0.001.
[2] Not assessed for snacks in the MDHQ; the intake for snacks was 0 g/day for all participants. [3] Not assessed for breakfast in the MDHQ; the intake for breakfast was 0 g/day for all participants. [4] Only assessed for breakfast in the MDHQ; the intake for all other eating occasions was 0 g/day for all participants.

Table S3. Mean estimates of daily intakes of Tier 1 food groups (in grams per day) derived from the 4-day weighed dietary record (DR) and those derived from the web version of the Meal-based Diet History Questionnaire (MDHQ) in 111 Japanese men, according to meal type [1].

	Breakfast		Lunch		Dinner		Snacks		Overall diet	
**Tier 1 food group**	**DR**	**Web MDHQ**	**DR**	**Web MDHQ**	**DR**	**Web MDHQ**	**DR**	**Web MDHQ**	**DR**	**Web MDHQ**
Rice [2]	62 ± 73	60 ± 62	122 ± 79	124 ± 67	138 ± 87	126 ± 61	0 ± 2	0 ± 0	322 ± 142	310 ± 103
Bread [2]	24 ± 29	27 ± 29	9 ± 18	11 ± 20	1 ± 5	1 ± 4	2 ± 6	0 ± 0**	36 ± 34	40 ± 36
Noodles [2]	4 ± 20	2 ± 5	81 ± 75	42 ± 40***	20 ± 36	27 ± 26	1 ± 4	0 ± 0	106 ± 82	70 ± 51***
Miso soup [2]	44 ± 67	44 ± 61	14 ± 31	25 ± 42**	60 ± 68	64 ± 58	0 ± 0	0 ± 0	119 ± 102	133 ± 102
Potatoes [2]	4 ± 8	4 ± 8	10 ± 14	5 ± 6***	22 ± 25	16 ± 9*	1 ± 2	0 ± 0**	36 ± 32	25 ± 17***
Pulses and nuts [2]	15 ± 27	9 ± 12**	6 ± 15	5 ± 7	31 ± 27	20 ± 13***	1 ± 3	0 ± 0***	52 ± 48	34 ± 22***
Vegetables [2]	31 ± 49	25 ± 31	56 ± 40	37 ± 33***	153 ± 77	107 ± 55***	2 ± 10	0 ± 0	241 ± 118	169 ± 84***
Pickled vegetables [2]	1 ± 4	3 ± 6*	3 ± 5	4 ± 7*	3 ± 10	5 ± 7	0 ± 1	0 ± 0	8 ± 15	12 ± 13*
Fruit	23 ± 40	15 ± 25**	9 ± 21	7 ± 14	10 ± 16	12 ± 19	10 ± 34	12 ± 23	51 ± 62	46 ± 53
Fish and shellfish [2]	5 ± 11	3 ± 5**	13 ± 16	10 ± 10	43 ± 33	28 ± 13***	1 ± 5	0 ± 0*	62 ± 40	41 ± 20***
Meat [2]	11 ± 15	8 ± 8*	32 ± 22	22 ± 12***	72 ± 40	42 ± 15***	1 ± 2	0 ± 0**	115 ± 47	71 ± 25***
Eggs [2]	17 ± 18	12 ± 13***	16 ± 16	10 ± 9***	11 ± 12	7 ± 5***	1 ± 2	0 ± 0**	45 ± 25	29 ± 17***
Dairy products	54 ± 85	42 ± 54	14 ± 38	10 ± 21	13 ± 30	7 ± 11*	26 ± 47	12 ± 26**	107 ± 122	71 ± 71***
Confectioneries	9 ± 18	5 ± 12**	7 ± 16	7 ± 14	3 ± 8	6 ± 15*	15 ± 21	40 ± 40***	35 ± 35	58 ± 52***
Fruit and vegetable juice	8 ± 34	18 ± 56*	4 ± 18	5 ± 20	4 ± 31	10 ± 41*	4 ± 27	9 ± 32	20 ± 68	41 ± 89***
Alcoholic beverages [3]	1 ± 3	0 ± 0	10 ± 47	10 ± 37	175 ± 304	216 ± 321	77 ± 207	70 ± 153	262 ± 394	297 ± 385
Soft drinks	2 ± 13	6 ± 21	11 ± 45	9 ± 26	8 ± 55	7 ± 23	71 ± 140	52 ± 86	92 ± 193	73 ± 119
Green tea	30 ± 71	47 ± 72**	47 ± 70	67 ± 88*	30 ± 69	41 ± 75	96 ± 178	104 ± 169	204 ± 284	258 ± 328
Barley tea	23 ± 52	37 ± 69*	65 ± 118	67 ± 89	47 ± 86	62 ± 84	106 ± 219	131 ± 193	240 ± 354	296 ± 376
Oolong tea	2 ± 14	13 ± 45**	10 ± 39	15 ± 49	10 ± 52	17 ± 56	24 ± 111	36 ± 120	46 ± 193	81 ± 237
Black tea	3 ± 15	10 ± 41*	2 ± 11	7 ± 29*	2 ± 13	6 ± 31	16 ± 53	20 ± 73	24 ± 66	42 ± 141
Coffee	82 ± 98	110 ± 94***	27 ± 47	58 ± 81***	8 ± 28	18 ± 45**	163 ± 218	187 ± 192	280 ± 273	372 ± 297***
Water	18 ± 42	67 ± 86***	19 ± 43	85 ± 98***	12 ± 40	89 ± 98***	181 ± 397	200 ± 218	229 ± 426	441 ± 410***
Breakfast cereals [4]	1 ± 5	2 ± 10	0 ± 0	0 ± 0	0 ± 0	0 ± 0	0 ± 0	0 ± 0	1 ± 5	2 ± 10

[1] Values are means ± standard deviations. The values derived from the MDHQ were compared with those derived from the DR using the paired *t*-test: * $p < 0.05$, ** $p < 0.01$, *** $p < 0.001$.
[2] Not assessed for snacks in the MDHQ; the intake for snacks was 0 g/day for all participants. [3] Not assessed for breakfast in the MDHQ; the intake for breakfast was 0 g/day for all participants. [4] Only assessed for breakfast in the MDHQ; the intake for all other eating occasions was 0 g/day for all participants.

Table S4. Bland–Altman analysis for estimates of daily intakes of Tier 1 food groups (in grams per day) derived from the 4-day weighed dietary record and those derived from the web version of the Meal-based Diet History Questionnaire (MDHQ) in 111 Japanese women, according to meal type [1].

	Breakfast			Lunch			Dinner			Snacks			Overall diet		
**Tier 1 food group**	**Mean difference**	**Limits of agreement**	**Slope of bias**	**Mean difference**	**Limits of agreement**	**Slope of bias**	**Mean difference**	**Limits of agreement**	**Slope of bias**	**Mean difference**	**Limits of agreement**	**Slope of bias**	**Mean difference**	**Limits of agreement**	**Slope of bias**
Rice [2]	1	−84, 87	−0.34***	2	−97, 102	−0.24*	−1	−92, 89	−0.30**	−1	−9, 7	----	2	−168, 172	−0.31***
Bread [2]	1	−48, 50	−0.20	−1	−37, 35	−0.78***	0	−12, 13	0.40**	−1	−8, 6	----	−1	−67, 66	−0.20
Noodles [2]	−1	−25, 22	−1.54***	−19	−118, 80	−0.74***	7	−47, 61	−0.60***	0	−3, 3	----	−14	−127, 100	−0.73***
Miso soup [2]	11	−59, 81	0.02	10	−65, 85	0.27*	13	−82, 108	0.01	0	−7, 6	----	33	−116, 183	0.17*
Potatoes [2]	2	−11, 15	0.34**	−4	−33, 25	−1.13***	−2	−37, 33	−0.97***	−1	−8, 6	----	−5	−55, 45	−0.51***
Pulses and nuts [2]	−5	−59, 49	−1.05***	−4	−45, 38	−1.44***	−4	−58, 51	−0.99***	−5	−37, 27	----	−17	−116, 82	−1.04***
Vegetables [2]	2	−65, 68	−0.25*	−19	−111, 74	−0.49***	−25	−150, 100	−0.42**	−2	−17, 13	----	−44	−255, 167	−0.53***
Pickled vegetables	2	−8, 12	0.54***	1	−12, 14	0.34	3	−18, 24	−0.38*	0	0, 0	----	5	−23, 33	0.12
Fruit	−8	−67, 51	−0.46***	−5	−42, 32	−0.66***	4	−36, 44	−0.17	1	−61, 64	−0.26	−8	−125, 110	−0.41***
Fish and shellfish [2]	−2	−19, 15	−0.88***	−6	−38, 26	−1.11***	−8	−49, 33	−0.90***	0	−6, 5	----	−16	−73, 40	−0.85***
Meat [2]	0	−20, 20	−0.60***	−10	−48, 29	−0.83***	−15	−77, 47	−1.13***	0	−4, 4	----	−25	−103, 54	−0.72***
Eggs [2]	−1	−27, 25	−0.49***	−4	−27, 19	−0.83***	−3	−22, 16	−0.97***	−1	−5, 4	----	−9	−48, 30	−0.53***
Dairy products	−1	−126, 124	−0.48***	−6	−79, 67	−0.81***	3	−35, 41	0.29*	−16	−103, 72	−0.66***	−20	−190, 150	−0.44***
Confectioneries	0	−29, 29	−0.37**	−1	−33, 30	−0.35*	2	−19, 22	0.82***	28	−31, 87	0.78***	28	−51, 107	0.51***
Fruit and vegetable juice	4	−47, 55	0.45***	0	−32, 32	−0.36***	2	−16, 21	0.65***	6	−55, 67	0.55***	12	−90, 115	0.49***
Alcoholic beverages [3]	0	−1, 1	----	1	−24, 26	1.98***	−6	−206, 193	−0.13*	3	−135, 140	−0.04	−3	−211, 205	−0.07
Soft drinks	5	−32, 41	1.31***	2	−40, 43	−0.15	2	−45, 50	0.23	0	−112, 112	0.07	8	−159, 176	0.46***
Green tea	25	−95, 144	0.31**	15	−114, 145	0.32**	12	−124, 148	0.08	28	−274, 330	−0.11	81	−410, 572	0.22*
Barley tea	16	−119, 151	−0.11	22	−111, 154	0.07	24	−146, 193	−0.12	25	−350, 400	−0.28*	87	−523, 696	−0.07
Oolong tea	4	−41, 50	1.98***	3	−55, 61	0.78***	5	−62, 72	0.12	12	−149, 174	0.71***	25	−231, 281	0.75***
Black tea	1	−60, 62	−0.08	0	−60, 60	0.12	5	−50, 60	0.86***	15	−127, 156	0.33***	21	−194, 235	0.47***
Coffee	33	−112, 178	−0.07	26	−90, 142	0.29**	15	−70, 99	1.02***	36	−174, 246	0.12	110	−229, 448	0.29***
Water	54	−97, 205	1.07***	57	−105, 219	1.55***	53	−112, 218	1.70***	45	−445, 535	−0.44***	209	−457, 876	0.36***
Breakfast cereals [4]	2	−11, 15	0.80***	0	−4, 3	----	0	−5, 5	----	0	0, 0	----	1	−13, 15	0.67***

[1] Mean difference was calculated as mean of difference between the two methods (MDHQ minus DR). Limits of agreement were calculated as the mean difference ± 1.96 standard deviation. Slope of bias (regression coefficient) was calculated by using a linear regression analysis, in which the difference between the two methods (MDHQ minus DR) was the dependent variable and the average of the two methods was the independent variable; positive values indicate that the difference increases as the average intake increases, while negative values indicate that the difference decreases as the average intake increases (* $p<0.05$, ** $p<0.01$, *** $p<0.001$). For the food groups the intake of which was not assessed in the MDHQ and thus the intake value was 0 g/day for all participants, the slope of bias was not calculated. [2] Not assessed for snacks in the MDHQ. [3] Not assessed for breakfast in the MDHQ. [4] Only assessed for breakfast in the MDHQ.

Table S5. Bland–Altman analysis for estimates of daily intakes of Tier 1 food groups (in grams per day) derived from the 4-day weighed dietary record (DR) and those derived from the web version of the Meal-based Diet History Questionnaire (MDHQ) in 111 Japanese men, according to meal type [1].

	Breakfast			Lunch			Dinner			Snacks			Overall diet		
**Tier 1 food group**	**Mean difference**	**Limits of agreement**	**Slope of bias**	**Mean difference**	**Limits of agreement**	**Slope of bias**	**Mean difference**	**Limits of agreement**	**Slope of bias**	**Mean difference**	**Limits of agreement**	**Slope of bias**	**Mean difference**	**Limits of agreement**	**Slope of bias**
Rice [2]	−2	−98, 95	−0.18*	2	−131, 135	−0.21*	−12	−144, 120	−0.42***	0	−5, 4	–––	−12	−240, 216	−0.40***
Bread [2]	4	−40, 47	0.03	3	−43, 48	0.18	0	−12, 12	−0.16	−2	−14, 10	–––	4	−62, 70	0.09
Noodles [2]	−3	−38, 32	−1.41***	−39	−171, 94	−0.82***	6	−76, 89	−0.59***	−1	−9, 8	–––	−36	−199, 128	−0.70***
Miso soup [2]	0	−85, 86	−0.1	11	−70, 92	0.45***	4	−98, 105	−0.18*	0	0, 0	–––	14	−164, 193	0.00
Potatoes [2]	0	−17, 17	0.08	−6	−34, 22	−1.31***	−5	−55, 44	−1.38***	−1	−4, 3	–––	−11	−76, 54	−0.96***
Pulses and nuts [2]	−6	−50, 38	−0.92***	0	−29, 28	−1.09***	−11	−66, 43	−1.11***	−1	−8, 6	–––	−19	−102, 65	−0.96***
Vegetables [2]	−6	−84, 72	−0.55***	−19	−103, 65	−0.29*	−46	−208, 116	−0.53***	−2	−21, 17	–––	−73	−297, 151	−0.47***
Pickled vegetables	1	−10, 13	0.57***	2	−13, 17	0.5**	2	−21, 24	−0.73***	0	−3, 2	–––	4	−30, 39	−0.2
Fruit	−8	−60, 44	−0.52***	−2	−38, 34	−0.56***	3	−39, 44	0.20	2	−65, 69	−0.59***	−6	−105, 94	0.0002*
Fish and shellfish [2]	−3	−20, 15	−0.99***	−3	−37, 31	−0.70***	−14	−79, 50	−1.30***	−1	−11, 9	–––	−21	−100, 58	−1.03***
Meat [2]	−3	−28, 22	−0.73***	−10	−53, 32	−0.82***	−30	−110, 50	−1.34***	−1	−5, 4	–––	−44	−139, 51	−0.97***
Eggs [2]	−5	−32, 22	−0.4***	−6	−35, 23	−0.80***	−4	−26, 18	−1.11***	−1	−5, 4	–––	−15	−55, 24	−0.47***
Dairy products	−12	−140, 117	−0.53***	−4	−67, 59	−0.74***	−6	−68, 55	−1.45***	−14	−99, 72	−0.81***	−36	−220, 149	−0.63***
Confectioneries	−5	−40, 31	−0.6***	0	−35, 35	−0.23	3	−31, 37	1.08***	25	−51, 102	0.88***	24	−82, 129	0.59***
Fruit and vegetable juice	10	−71, 91	0.58***	1	−33, 35	0.14	6	−51, 63	0.33***	5	−71, 80	0.28	21	−106, 148	0.31***
Alcoholic beverages [3]	−1	−7, 5	–––	0	−78, 78	−0.31**	41	−407, 490	0.06	−6	−334, 321	−0.37**	35	−362, 431	−0.02
Soft drinks	3	−39, 45	0.74***	−2	−76, 73	−0.67***	−1	−117, 115	−1.36***	−19	−273, 234	−0.66***	−18	−350, 314	−0.63***
Green tea	17	−95, 129	0.02	20	−167, 206	0.34*	10	−147, 168	0.12	7	−368, 383	−0.07	54	−534, 643	0.19
Barley tea	14	−125, 153	0.43**	2	−221, 225	−0.39**	15	−167, 196	−0.05	25	−366, 416	−0.16	56	−584, 696	0.08
Oolong tea	10	−69, 89	1.3***	6	−84, 96	0.29*	7	−87, 100	0.08	12	−232, 255	0.11	35	−366, 435	0.26*
Black tea	6	−58, 70	1.06***	5	−43, 52	1.08***	3	−51, 58	1.04***	4	−148, 157	0.51***	19	−232, 269	0.97***
Coffee	27	−130, 184	−0.05	31	−94, 156	0.64***	10	−68, 88	0.61***	23	−302, 348	−0.15	92	−313, 498	0.10
Water	49	−129, 227	1.1***	66	−127, 259	1.18***	77	−121, 274	1.31***	20	−725, 764	−0.83***	212	−624, 1047	−0.05
Breakfast cereals [4]	1	−13, 15	0.79***	0	0, 0	–––	0	0, 0	–––	0	0, 0	–––	1	−13, 15	0.79***

[1] Mean difference was calculated as mean of difference between the two methods (MDHQ minus DR). Limits of agreement were calculated as the mean difference ± 1.96 standard deviation. Slope of bias (regression coefficient) was calculated by using a linear regression analysis, in which the difference between the two methods (MDHQ minus DR) was the dependent variable and the average of the two methods was the independent variable; positive values indicate that the difference increases as the average intake increases, while negative values indicate that the difference decreases as the average intake increases (* p <0.05, ** p <0.01, *** p <0.001). For the food groups the intake of which was not assessed in the MDHQ and thus the intake value was 0 g/day for all participants, the slope of bias was not calculated. [2] Not assessed for snacks in the MDHQ. [3] Not assessed for breakfast in the MDHQ. [4] Only assessed for breakfast in the MDHQ.

Table S6. Mean estimates of overall intakes of Tier 2 food groups (in grams per day) derived from the 4-day weighed dietary record (DR) and those derived from the web version of the Meal-based Diet History Questionnaire (MDHQ) and Bland–Altman analysis for these estimates in 111 Japanese women and 111 Japanese men [1].

	Women					Men				
			Bland–Altman analysis [2]					Bland–Altman analysis [2]		
Tier 2 food group	DR	Web MDHQ	Mean difference	Limits of agreement	Slope of bias	DR	Web MDHQ	Mean difference	Limits of agreement	Slope of bias
Rice										
White rice	206 ± 110	202 ± 84	−4	−179, 171	−0.34***	320 ± 144	293 ± 106*	−27	−265, 211	−0.39***
Brown rice	3 ± 18	8 ± 19**	6	−31, 42	0.10	2 ± 14	17 ± 44***	15	−62, 92	1.31***
Bread										
White bread	35 ± 31	29 ± 24	−6	−69, 58	−0.37**	35 ± 34	35 ± 32	0	−65, 64	−0.09
Wholegrain bread	1 ± 5	6 ± 9***	5	−12, 22	0.86***	1 ± 5	5 ± 9***	5	−15, 24	1.06***
Noodles										
Wheat noodles	17 ± 34	33 ± 24***	16	−61, 93	−0.60***	26 ± 51	35 ± 27	9	−97, 115	−1.03***
Chinese noodles	18 ± 36	10 ± 12*	−8	−76, 59	−1.33***	26 ± 45	20 ± 32	−6	−109, 97	−0.60***
Instant noodles	13 ± 27	2 ± 6***	−10	−58, 38	−1.50***	30 ± 51	4 ± 9***	−26	−120, 68	−1.63***
Spaghetti	15 ± 23	7 ± 9***	−9	−54, 36	−1.26***	16 ± 31	7 ± 12***	−10	−67, 47	−1.18***
Buckwheat noodles	6 ± 19	4 ± 7	−2	−38, 34	−1.26***	8 ± 23	4 ± 7	−4	−48, 41	−1.47***
Pulses and nuts										
Soy milk	14 ± 41	1 ± 2***	−13	−91, 65	−1.90***	3 ± 14	0 ± 1	−2	−30, 25	−1.93***
Tofu (i.e., soybean curd)	25 ± 26	31 ± 19*	6	−51, 63	−0.47**	31 ± 38	25 ± 18	−5	−74, 64	−0.99***
Natto (i.e., fermented soybeans)	8 ± 11	6 ± 6**	−2	−19, 14	−0.64***	6 ± 11	5 ± 7*	−2	−19, 15	−0.54***
Tofu products	4 ± 8	4 ± 4	0	−15, 14	−1.03***	6 ± 11	3 ± 4**	−3	−25, 18	−1.47***
Peanuts and nuts	5 ± 12	0 ± 1***	−5	−29, 19	−1.97***	5 ± 9	0 ± 1***	−4	−22, 13	−1.88***
All other pulses and nuts	2 ± 5	0 ± 0***	−2	−12, 8	−1.94***	2 ± 5	1 ± 1*	−1	−12, 9	−1.84***
Vegetables										
Edamame (i.e., immature soybeans) and peas	3 ± 8	1 ± 2	−1	−18, 15	−1.76***	2 ± 6	2 ± 2	−1	−12, 10	−1.26***
Seaweeds	6 ± 7	6 ± 5	−1	−17, 15	−0.55***	7 ± 11	6 ± 7	−1	−25, 24	−0.82***
Pumpkins	7 ± 11	4 ± 4**	−3	−23, 17	−1.18***	7 ± 13	3 ± 4***	−4	−27, 19	−1.31***
Mushrooms	13 ± 14	9 ± 7**	−4	−30, 22	−1.00***	13 ± 13	8 ± 9***	−6	−31, 20	−0.57***
Cabbage	26 ± 25	18 ± 13***	−9	−61, 44	−0.99***	35 ± 29	21 ± 19***	−13	−73, 46	−0.63***
Cucumbers	13 ± 13	12 ± 8	−1	−31, 29	−0.86***	12 ± 13	11 ± 9	−1	−28, 26	−0.53***
Bitter melon	2 ± 7	1 ± 1**	−2	−14, 10	−1.58***	2 ± 6	1 ± 2*	−1	−12, 9	−1.40***
Burdock	2 ± 5	1 ± 1***	−2	−11, 7	−1.75***	4 ± 6	1 ± 1***	−3	−16, 9	−1.92***
Radishes	12 ± 15	8 ± 9*	−3	−32, 25	−0.73***	12 ± 15	9 ± 10	−3	−38, 32	−0.81***
Onions	27 ± 21	27 ± 17	−1	−47, 46	−0.29***	33 ± 37	28 ± 20	−5	−79, 69	−0.92***
Chinese cabbage	3 ± 6	1 ± 3	−1	−14, 12	−1.28***	3 ± 8	2 ± 5	−1	−17, 16	−0.80***
Tomatoes	16 ± 20	25 ± 18***	9	−36, 54	−0.18	18 ± 38	24 ± 22	6	−73, 85	−0.87***
Eggplants	10 ± 19	9 ± 7	−1	−35, 33	−1.20***	11 ± 23	8 ± 7	−4	−48, 41	−1.48***
Carrots	13 ± 14	15 ± 11	2	−25, 29	−0.32*	14 ± 15	16 ± 14	2	−29, 32	−0.08
Green peppers	5 ± 8	4 ± 3	−1	−16, 14	−1.14***	7 ± 10	3 ± 3***	−4	−23, 15	−1.39***
Broccoli	4 ± 9	3 ± 3	−2	−18, 15	−1.24***	5 ± 9	2 ± 3**	−2	−18, 14	−1.19***

Table S6. Continued

Tier 2 food group	Women DR	Women Web MDHQ	Women Bland–Altman analysis [2] Mean difference	Women Bland–Altman analysis [2] Limits of agreement	Women Bland–Altman analysis [2] Slope of bias	Men DR	Men Web MDHQ	Men Bland–Altman analysis [2] Mean difference	Men Bland–Altman analysis [2] Limits of agreement	Men Bland–Altman analysis [2] Slope of bias
Green leafy vegetables	14 ± 19	16 ± 13	2	−41, 44	−0.64***	14 ± 15	12 ± 12	−1	−37, 34	−0.32
Bean sprouts	10 ± 13	4 ± 4***	−6	−32, 19	−1.53***	12 ± 16	4 ± 5***	−8	−36, 20	−1.34***
Lettuce	7 ± 9	5 ± 5*	−2	−20, 16	−0.91***	8 ± 18	5 ± 6	−3	−35, 30	−1.32***
All other vegetables	19 ± 19	1 ± 1***	−18	−55, 19	−1.97***	22 ± 21	2 ± 2***	−20	−62, 21	−2.02***
Fruit										
Strawberries	0 ± 0	0 ± 1*	0	−2, 3	1.94***	0 ± 0	0 ± 2*	0	−3, 4	1.88***
Persimmons	0 ± 2	0 ± 1	0	−5, 5	−0.60**	1 ± 4	0 ± 0	−1	−8, 7	−1.85***
Citrus	5 ± 13	10 ± 19*	4	−39, 48	0.70***	3 ± 10	7 ± 16*	4	−30, 39	0.83***
Kiwi fruit	2 ± 6	1 ± 3	−1	−9, 8	−0.88***	2 ± 6	1 ± 2*	−1	−10, 8	−1.24***
Watermelon	2 ± 10	1 ± 3	−1	−21, 19	−1.76***	1 ± 6	1 ± 2	0	−13, 12	−1.72***
Pears	11 ± 20	6 ± 10*	−5	−44, 35	−0.94***	10 ± 22	5 ± 10**	−6	−46, 35	−1.05***
Bananas	12 ± 21	17 ± 27*	5	−43, 53	0.33**	12 ± 26	14 ± 29	2	−49, 54	0.16
Grapes	15 ± 31	6 ± 10**	−9	−64, 46	−1.30***	12 ± 32	3 ± 7**	−8	−65, 48	−1.50***
Melon	2 ± 15	1 ± 3	−1	−30, 28	−1.81***	0 ± 1	1 ± 2**	1	−4, 5	0.61***
Peaches	2 ± 9	3 ± 7	1	−18, 20	−0.48**	2 ± 11	1 ± 2	−1	−21, 19	−1.70***
Apples	6 ± 15	6 ± 14	1	−22, 24	−0.07	4 ± 13	7 ± 18*	3	−25, 30	0.35***
All other fruit	10 ± 30	1 ± 1**	−9	−68, 50	−2.00***	4 ± 10	2 ± 4**	−3	−25, 19	−1.54***
Fish and shellfish										
Oily fish	12 ± 20	7 ± 7**	−5	−39, 30	−1.18***	14 ± 19	9 ± 8**	−5	−43, 33	−1.22***
Red meat fish	5 ± 8	5 ± 5	0	−17, 18	−0.86***	5 ± 10	7 ± 6	2	−21, 24	−0.77***
Squid and octopus	3 ± 5	1 ± 2*	−1	−11, 9	−1.20***	4 ± 7	2 ± 3**	−2	−15, 11	−1.24***
Eel	0 ± 2	0 ± 0	0	−4, 3	−1.92***	1 ± 4	0 ± 1	−1	−9, 7	−1.98***
Shrimp and crab	3 ± 5	1 ± 2**	−1	−12, 9	−1.60***	4 ± 7	2 ± 3**	−2	−17, 13	−1.49***
Shellfish	1 ± 3	1 ± 1	0	−7, 6	−1.24***	1 ± 3	1 ± 2	0	−7, 7	−0.94***
Small fish with bones	1 ± 2	0 ± 1	0	−3, 3	−1.21***	1 ± 3	0 ± 1	0	−5, 5	−1.70***
Fish eggs	1 ± 2	0 ± 0**	−1	−5, 4	−1.70***	1 ± 3	0 ± 0**	−1	−7, 5	−1.96***
Dried fish	4 ± 10	2 ± 2*	−2	−22, 18	−1.73***	5 ± 13	2 ± 4*	−3	−28, 22	−1.62***
Salmon	8 ± 11	4 ± 4***	−4	−27, 19	−1.40***	9 ± 13	6 ± 7*	−4	−33, 26	−1.24***
White meat fish	5 ± 11	4 ± 5	−2	−24, 20	−1.24***	7 ± 13	5 ± 6	−1	−28, 25	−1.22***
Ground fish meat products	5 ± 7	5 ± 5	0	−15, 16	−0.71***	8 ± 14	5 ± 6*	−3	−32, 26	−1.27***
Canned tuna	2 ± 3	1 ± 1**	−1	−7, 5	−1.33***	2 ± 4	1 ± 2*	−1	−10, 8	−1.09***
All other fish and shellfish	0 ± 0	0 ± 0	0	0, 0	−1.73***	0 ± 1	0 ± 0	0	−2, 2	−1.99***
Meat										
Beef	13 ± 17	7 ± 6***	−5	−38, 27	−1.33***	17 ± 22	10 ± 8**	−7	−49, 35	−1.35***
Chicken	30 ± 26	16 ± 10***	−14	−66, 37	−1.31***	46 ± 39	19 ± 11***	−26	−104, 51	−1.64***
Processed meat	10 ± 10	10 ± 8	0	−20, 20	−0.36**	13 ± 13	13 ± 11	0	−29, 29	−0.30*
Pork	30 ± 25	26 ± 14	−4	−56, 48	−0.95***	39 ± 27	29 ± 14***	−10	−70, 50	−1.14***

Table S6. Continued

Tier 2 food group	Women DR	Women Web MDHQ	Women Bland–Altman analysis [2] Mean difference	Women Bland–Altman analysis [2] Limits of agreement	Women Bland–Altman analysis [2] Slope of bias	Men DR	Men Web MDHQ	Men Bland–Altman analysis [2] Mean difference	Men Bland–Altman analysis [2] Limits of agreement	Men Bland–Altman analysis [2] Slope of bias
Liver	0 ± 2	0 ± 0	0	−5, 5	−1.92***	1 ± 4	0 ± 0	0	−8, 7	−1.95***
All other meat	0 ± 1	0 ± 0	0	−3, 3	−2.00***	0 ± 3	0 ± 0	0	−5, 5	−2.00***
Dairy products										
Ice cream	6 ± 13	6 ± 15	0	−34, 34	0.24	6 ± 14	5 ± 11	−1	−35, 34	−0.38*
Cheese	5 ± 7	5 ± 10	0	−23, 23	0.79***	5 ± 7	4 ± 14	−1	−31, 30	1.23***
Low-fat milk	10 ± 35	5 ± 16	−6	−64, 53	−0.95***	18 ± 77	7 ± 27	−11	−127, 105	−1.06***
Yogurt	67 ± 93	38 ± 54***	−29	−174, 115	−0.65***	53 ± 85	28 ± 52***	−25	−147, 97	−0.57***
Full-fat milk	38 ± 56	56 ± 57**	18	−98, 133	0.03	20 ± 42	25 ± 41	5	−66, 77	−0.04
All other dairy products	4 ± 11	1 ± 4*	−3	−25, 19	−1.32***	5 ± 20	2 ± 7	−3	−43, 36	−1.39***
Confectioneries										
Candies, caramels, and chewing gum	0 ± 2	1 ± 2	0	−4, 4	−0.04	0 ± 1	1 ± 2**	1	−4, 6	1.54***
Japanese bread with a sweet filling	3 ± 9	7 ± 10***	4	−20, 28	0.15	8 ± 15	7 ± 15	−1	−38, 36	0.09
Snacks made from wheat flour	1 ± 4	4 ± 8***	3	−12, 18	1.01***	2 ± 8	4 ± 7**	3	−15, 20	−0.19
Jellies	4 ± 11	1 ± 1***	−3	−24, 17	−1.85***	4 ± 14	1 ± 2**	−4	−31, 24	−1.91***
Rice crackers	2 ± 5	8 ± 11***	6	−14, 26	1.12***	1 ± 4	9 ± 13***	7	−18, 32	1.55***
Chocolates	4 ± 6	12 ± 15***	8	−20, 36	1.13***	3 ± 5	10 ± 16***	7	−21, 36	1.33***
Biscuits and cookies	2 ± 4	4 ± 6***	2	−10, 14	0.39*	1 ± 3	2 ± 4	1	−8, 10	0.18
Cakes	9 ± 17	16 ± 16***	7	−34, 47	−0.05	8 ± 17	14 ± 17**	6	−31, 43	0.06
Japanese sweets	8 ± 14	10 ± 12	1	−31, 34	−0.25	7 ± 16	11 ± 20	3	−44, 50	0.38*
Alcoholic beverages										
Beer	55 ± 140	61 ± 149	5	−135, 146	0.07	174 ± 329	207 ± 332*	33	−288, 355	0.01
Sake	1 ± 9	2 ± 14	1	−11, 13	0.38***	8 ± 35	10 ± 39	3	−63, 68	0.12
Shochu (i.e., Japanese distilled beverages)	23 ± 69	20 ± 63	−4	−112, 104	−0.10	65 ± 153	64 ± 112	−1	−252, 250	−0.39***
Wine	10 ± 61	6 ± 20	−3	−101, 94	−1.16***	6 ± 32	9 ± 51	3	−99, 105	0.69***
Whiskey and other spirits	0 ± 0	0 ± 3	0	−6, 7	2.00***	6 ± 27	6 ± 30	0	−50, 49	0.11

[1] Values are means ± standard deviations. The values derived from the MDHQ were compared with those derived from the DR using the paired *t*-test: * *p* <0.05, ** *p* <0.01, *** *p* <0.001. [2] Mean difference was calculated as mean of difference between the two methods (MDHQ minus DR). Limits of agreement were calculated as the mean difference ± 1.96 standard deviation. Slope of bias (regression coefficient) was calculated by using a linear regression analysis, in which the difference between the two methods (MDHQ minus DR) was the dependent variable and the average of the two methods was the independent variable; positive values indicate that the difference increases as the average intake increases, while negative values indicate that the difference decreases as the average intake increases (* *p* <0.05, ** *p* <0.01, *** *p* <0.001).

Table S7. Median estimates of daily intakes of Tier 1 food groups (in grams per day) derived from the paper version of the Meal-based Diet History Questionnaire (MDHQ) in 111 Japanese women and 111 Japanese men, according to meal type [1].

	Women					Men				
**Tier 1 food group**	**Breakfast**	**Lunch**	**Dinner**	**Snacks**	**Overall diet**	**Breakfast**	**Lunch**	**Dinner**	**Snacks**	**Overall diet**
Rice [2]	35 (0, 89)	82 (41, 104)	97 (39, 116)	0 (0, 0)**	220 (155, 258)	48 (11, 121)	122 (86, 173)	140 (83, 171)*	0 (0, 0)*	317 (255, 383)
Bread [2]	19 (5, 45)	10 (0, 20)*	0 (0, 0)	0 (0, 0)**	38 (15, 56)	12 (0, 46)	6 (0, 13)*	0 (0, 0)	0 (0, 0)***	30 (11, 64)
Noodles [2]	0 (0, 0)	25 (13, 50)*	10 (10, 21)*	0 (0, 0)	46 (29, 78)	0 (0, 0)	26 (13, 53)***	22 (0, 25)	0 (0, 0)	51 (34, 92)***
Miso soup [2]	13 (0, 100)***	12 (0, 71)***	75 (13, 123)***	0 (0, 0)	140 (75, 217)***	13 (0, 100)	11 (0, 67)***	52 (13, 127)**	0 (0, 0)	146 (63, 231)***
Potatoes [2]	3 (0, 6)*	5 (3, 6)*	14 (7, 21)	0 (0, 0)***	21 (13, 28)**	0 (0, 6)	3 (0, 5)***	8 (8, 17)**	0 (0, 0)***	19 (9, 28)***
Pulses and nuts [2]	11 (0, 22)	6 (3, 11)	16 (8, 30)	0 (0, 0)***	39 (21, 58)*	3 (0, 16)*	3 (0, 11)	18 (9, 28)**	0 (0, 0)***	31 (18, 52)***
Vegetables [2]	24 (6, 59)*	40 (13, 66)**	117 (75, 138)***	0 (0, 0)***	171 (122, 237)***	12 (0, 46)	40 (7, 64)***	116 (69, 154)***	0 (0, 0)***	177 (103, 240)***
Pickled vegetables [2]	0 (0, 7)***	0 (0, 3)*	2 (0, 7)***	0 (0, 0)	5 (0, 13)***	0 (0, 4)***	2 (0, 9)**	3 (0, 10)***	0 (0, 0)	12 (2, 21)***
Fruit	11 (0, 34)	5 (0, 20)	6 (0, 23)**	12 (0, 25)***	62 (23, 102)	6 (0, 23)*	0 (0, 5)	6 (0, 22)	8 (0, 30)***	30 (12, 86)
Fish and shellfish [2]	2 (0, 4)	6 (3, 13)**	19 (10, 27)***	0 (0, 0)**	29 (19, 40)***	2 (0, 5)	7 (4, 14)	26 (13, 37)***	0 (0, 0)**	40 (27, 55)***
Meat [2]	7 (2, 13)	16 (10, 22)***	34 (25, 43)***	0 (0, 0)**	59 (43, 73)***	8 (2, 12)	21 (14, 30)***	41 (31, 53)***	0 (0, 0)**	72 (56, 88)***
Eggs [2]	12 (0, 22)	8 (4, 12)*	7 (4, 10)*	0 (0, 0)***	28 (15, 38)**	12 (3, 25)	9 (2, 14)***	8 (4, 11)*	0 (0, 0)***	31 (17, 45)***
Dairy products	57 (18, 123)	6 (0, 24)	6 (0, 22)*	13 (0, 40)	112 (63, 180)	10 (0, 111)	0 (0, 7)	5 (0, 11)	0 (0, 13)**	43 (14, 134)***
Confectioneries	0 (0, 14)	4 (0, 8)	0 (0, 4)**	36 (18, 64)***	58 (32, 88)***	0 (0, 5)	0 (0, 5)	0 (0, 4)	23 (4, 54)***	39 (14, 73)***
Fruit and vegetable juice	0 (0, 0)*	0 (0, 0)	0 (0, 0)*	0 (0, 21)***	0 (0, 42)***	0 (0, 0)***	0 (0, 0)	0 (0, 0)*	0 (0, 18)***	0 (0, 52)***
Alcoholic beverages [3]	0 (0, 0)***	0 (0, 0)***	0 (0, 43)**	0 (0, 0)	0 (0, 70)**	0 (0, 0)***	0 (0, 0)*	66 (0, 251)	0 (0, 75)	132 (0, 377)
Soft drinks	0 (0, 0)***	0 (0, 0)	0 (0, 0)	8 (0, 34)	16 (0, 48)	0 (0, 0)**	0 (0, 11)	0 (0, 0)*	12 (0, 61)	22 (0, 103)
Green tea	0 (0, 51)**	14 (0, 84)	0 (0, 84)	36 (0, 153)	94 (0, 375)	0 (0, 52)*	15 (0, 101)	0 (0, 56)	29 (0, 185)*	146 (0, 417)*
Barley tea	0 (0, 105)***	57 (0, 165)***	56 (0, 168)***	97 (0, 271)***	266 (0, 677)***	0 (0, 49)***	17 (0, 101)	0 (0, 146)***	31 (0, 222)***	146 (0, 528)***
Oolong tea	0 (0, 0)**	0 (0, 0)*	0 (0, 0)	0 (0, 0)***	0 (0, 15)***	0 (0, 0)***	0 (0, 0)	0 (0, 0)	0 (0, 17)**	0 (0, 17)***
Black tea	0 (0, 0)	0 (0, 0)	0 (0, 0)*	0 (0, 40)**	13 (0, 79)**	0 (0, 0)***	0 (0, 0)**	0 (0, 0)	0 (0, 16)*	0 (0, 17)**
Coffee	148 (15, 205)***	25 (0, 80)***	0 (0, 0)*	128 (49, 261)***	333 (171, 475)***	122 (0, 208)***	15 (0, 119)***	0 (0, 0)**	144 (15, 363)***	298 (137, 626)***
Water	26 (0, 171)***	28 (0, 117)***	28 (0, 174)***	156 (24, 355)***	366 (73, 619)***	0 (0, 170)***	17 (0, 153)***	15 (0, 188)***	106 (15, 309)***	272 (15, 652)***
Breakfast cereals [4]	0 (0, 0)***	0 (0, 0)	0 (0, 0)	0 (0, 0)	0 (0, 0)**	0 (0, 0)**	0 (0, 0)	0 (0, 0)	0 (0, 0)	0 (0, 0)**

[1] Values are medians (25th and 75th percentiles). The values derived from the MDHQ were compared with those derived from the DR (shown in Table 2 for women and Table 3 for men) using the Wilcoxon signed-rank test: * $p < 0.05$, ** $p < 0.01$, *** $p < 0.001$. [2] Not assessed for snacks in the MDHQ; the intake for snacks was 0 g/day for all participants. [3] Not assessed for breakfast in the MDHQ; the intake for breakfast was 0 g/day for all participants. [4] Only assessed for breakfast in the MDHQ; the intake for all other eating occasions was 0 g/day for all participants.

Table S8. Mean estimates of daily intakes of Tier 1 food groups (in grams per day) derived from the paper version of the Meal-based Diet History Questionnaire (MDHQ) in 111 Japanese women and 111 Japanese men, according to meal type [1].

	Women					Men				
**Tier 1 food group**	**Breakfast**	**Lunch**	**Dinner**	**Snacks**	**Overall diet**	**Breakfast**	**Lunch**	**Dinner**	**Snacks**	**Overall diet**
Rice [2]	46 ± 44	78 ± 38	84 ± 44	0 ± 0*	209 ± 81	64 ± 61	125 ± 59	124 ± 64*	0 ± 0	313 ± 104
Bread [2]	25 ± 22	11 ± 12	1 ± 3	0 ± 0**	38 ± 25	25 ± 27	12 ± 17	1 ± 2	0 ± 0**	37 ± 32
Noodles [2]	2 ± 6	39 ± 32**	17 ± 15	0 ± 0	58 ± 38	3 ± 7	38 ± 34***	21 ± 19	0 ± 0	62 ± 41***
Miso soup [2]	50 ± 60***	36 ± 48***	70 ± 56***	0 ± 0	156 ± 106***	51 ± 62	32 ± 45***	70 ± 59*	0 ± 0	152 ± 110***
Potatoes [2]	4 ± 6	5 ± 5***	14 ± 8*	0 ± 0*	23 ± 14***	4 ± 6	5 ± 5***	13 ± 9***	0 ± 0**	22 ± 16***
Pulses and nuts [2]	13 ± 13	8 ± 9	21 ± 13	0 ± 0**	42 ± 26***	10 ± 13*	6 ± 7	21 ± 14***	0 ± 0***	36 ± 24***
Vegetables [2]	33 ± 30	43 ± 31***	101 ± 43***	0 ± 0**	177 ± 80***	26 ± 29	39 ± 31***	105 ± 52***	0 ± 0	170 ± 87***
Pickled vegetables [2]	3 ± 5***	3 ± 5	4 ± 5	0 ± 0	10 ± 13**	3 ± 6***	5 ± 6***	6 ± 6	0 ± 0	14 ± 14***
Fruit	21 ± 25*	11 ± 14	15 ± 19	20 ± 25**	67 ± 55	16 ± 23**	7 ± 11	13 ± 17	17 ± 23**	52 ± 49
Fish and shellfish [2]	3 ± 5	8 ± 7**	21 ± 12***	0 ± 0	32 ± 18***	4 ± 5	10 ± 8*	27 ± 13***	0 ± 0*	41 ± 20***
Meat [2]	7 ± 7	16 ± 11***	35 ± 14***	0 ± 0*	58 ± 23***	9 ± 8	22 ± 12***	42 ± 15***	0 ± 0**	73 ± 24***
Eggs [2]	13 ± 11	9 ± 6**	7 ± 5**	0 ± 0**	29 ± 15***	15 ± 14	10 ± 8***	8 ± 6**	0 ± 0**	33 ± 19***
Dairy products	67 ± 49	19 ± 25	12 ± 15	28 ± 39*	126 ± 89	43 ± 53	10 ± 19	10 ± 15	13 ± 27**	76 ± 77***
Confectioneries	7 ± 12	8 ± 13	4 ± 8*	44 ± 32***	64 ± 45***	6 ± 14	5 ± 10	4 ± 10	32 ± 32***	48 ± 42**
Fruit and vegetable juice	9 ± 25*	5 ± 14	4 ± 15*	15 ± 30**	33 ± 58**	16 ± 49*	7 ± 22	7 ± 32*	21 ± 47***	50 ± 87***
Alcoholic beverages [3]	0 ± 0**	1 ± 9	54 ± 125*	22 ± 77	77 ± 165	0 ± 0	10 ± 33	177 ± 255	81 ± 168	268 ± 353
Soft drinks	6 ± 19**	6 ± 18	4 ± 16	25 ± 40	41 ± 75	7 ± 22*	13 ± 32	8 ± 23	52 ± 85	80 ± 126
Green tea	40 ± 64**	49 ± 66	49 ± 74	100 ± 142	238 ± 301*	37 ± 65	58 ± 77	37 ± 68	117 ± 162	249 ± 310
Barley tea	51 ± 68***	76 ± 78***	79 ± 83**	160 ± 174**	365 ± 362***	37 ± 65**	65 ± 84	66 ± 86*	139 ± 188*	306 ± 359**
Oolong tea	7 ± 29*	13 ± 41*	10 ± 34	29 ± 80**	59 ± 163**	12 ± 38**	12 ± 41	15 ± 49	45 ± 126*	83 ± 236*
Black tea	16 ± 42	12 ± 34	6 ± 23*	39 ± 74*	74 ± 146**	11 ± 37**	7 ± 27*	4 ± 24	29 ± 92	51 ± 157*
Coffee	119 ± 88***	54 ± 65***	16 ± 43*	170 ± 152***	359 ± 263***	110 ± 91***	62 ± 79***	19 ± 47**	207 ± 200**	398 ± 324***
Water	73 ± 78***	66 ± 79***	69 ± 83***	208 ± 193*	416 ± 377***	58 ± 81***	71 ± 89***	78 ± 91***	197 ± 219	404 ± 411***
Breakfast cereals [4]	3 ± 10***	0 ± 0	0 ± 0	0 ± 0	3 ± 10*	3 ± 11*	0 ± 0	0 ± 0	0 ± 0	3 ± 11*

[1] Values are means ± standard deviations. The values derived from the MDHQ were compared with those derived from the DR (shown in Table S2 for women and Table S3 for men) using the paired *t*-test: * $p < 0.05$, ** $p < 0.01$, *** $p < 0.001$. [2] Not assessed for snacks in the MDHQ; the intake for snacks was 0 g/day for all participants. [3] Not assessed for breakfast in the MDHQ; the intake for breakfast was 0 g/day for all participants. [4] Only assessed for breakfast in the MDHQ; the intake for all other eating occasions was 0 g/day for all participants.

Table S9. Spearman correlation coefficients between estimates of daily intakes of Tier 1 food groups (in grams per day) derived from the 4-day weighed dietary record and those derived from the paper version of the Meal-based Diet History Questionnaire (MDHQ) in 111 Japanese women and 111 Japanese men, according to meal type [1].

	Women					Men				
**Tier 1 food group**	**Breakfast**	**Lunch**	**Dinner**	**Snacks**	**Overall diet**	**Breakfast**	**Lunch**	**Dinner**	**Snacks**	**Overall diet**
Rice [2]	0.84***	0.49***	0.65***	–––	0.65***	0.81***	0.64***	0.63***	–––	0.55***
Bread [2]	0.66***	0.31***	0.13	–––	0.61***	0.74***	0.64***	0.29**	–––	0.63***
Noodles [2]	0.22*	0.42***	0.23*	–––	0.28**	0.42***	0.48***	0.12	–––	0.31**
Miso soup [2]	0.79***	0.41***	0.78***	–––	0.70***	0.77***	0.37***	0.76***	–––	0.73***
Potatoes [2]	0.43***	0.12	0.14	–––	0.17	0.41***	0.23*	0.08	–––	0.22*
Pulses and nuts [2]	0.65***	0.40***	0.13	–––	0.49***	0.70***	0.22*	0.27**	–––	0.46***
Vegetables [2]	0.69***	0.36***	0.29**	–––	0.43***	0.67***	0.44***	0.24*	–––	0.48***
Pickled vegetables [2]	0.42***	0.35***	0.20*	–––	0.43***	0.30**	0.28**	0.20*	–––	0.34***
Fruit	0.66***	0.53***	0.46***	0.45***	0.70***	0.65***	0.34***	0.27**	0.39***	0.64***
Fish and shellfish [2]	0.57***	0.34***	0.27**	–––	0.32***	0.39***	0.24*	0.20*	–––	0.33***
Meat [2]	0.58***	0.37***	0.27**	–––	0.34***	0.53***	0.39***	0.17	–––	0.26**
Eggs [2]	0.50***	0.25**	0.30**	–––	0.42***	0.70***	0.48***	0.39***	–––	0.59***
Dairy products	0.77***	0.22*	0.33***	0.54***	0.69***	0.69***	0.31***	0.38***	0.23*	0.70***
Confectioneries	0.30**	0.25**	0.37***	0.50***	0.46***	0.39***	0.40***	0.02	0.53***	0.52***
Fruit and vegetable juice	0.36***	0.17	0.37***	0.35***	0.49***	0.58***	0.32***	0.25**	0.29**	0.47***
Alcoholic beverages [3]	–––	0.04	0.73***	0.48***	0.76***	–––	0.08	0.73***	0.64***	0.83***
Soft drinks	−0.03	0.32***	0.07	0.38***	0.44***	0.19*	0.12	0.22*	0.45***	0.43***
Green tea	0.57***	0.58***	0.57***	0.53***	0.70***	0.65***	0.51***	0.53***	0.65***	0.70***
Barley tea	0.63***	0.67***	0.73***	0.71***	0.79***	0.66***	0.58***	0.68***	0.63***	0.73***
Oolong tea	−0.03	0.39***	0.50***	0.38***	0.53***	0.46***	0.66***	0.54***	0.45***	0.42***
Black tea	0.74***	0.63***	0.33***	0.55***	0.70***	0.59***	0.60***	0.34***	0.34***	0.43***
Coffee	0.71***	0.52***	0.50***	0.73***	0.76***	0.75***	0.60***	0.47***	0.75***	0.77***
Water	0.35***	0.33***	0.17	0.63***	0.64***	0.27**	0.31***	0.36***	0.69***	0.63***
Breakfast cereals [4]	0.68***	–––	–––	–––	0.67***	0.52***	–––	–––	–––	0.44***

[1] Values are Spearman correlation coefficients. * $p<0.05$, ** $p<0.01$, *** $p<0.001$. [2] Not assessed for snacks in the MDHQ; the intake for snacks was 0 g/day for all participants. [3] Not assessed for breakfast in the MDHQ; the intake for breakfast was 0 g/day for all participants. [4] Only assessed for breakfast in the MDHQ; the intake for all other eating occasions was 0 g/day for all participants.

Table S10. Bland–Altman analysis for estimates of daily intakes of Tier 1 food groups (in grams per day) derived from the 4-day weighed dietary record (DR) and those derived from the paper version of the Meal-based Diet History Questionnaire (MDHQ) in 111 Japanese women, according to meal type [1].

	Breakfast			Lunch			Dinner			Snacks			Overall diet		
**Tier 1 food group**	**Mean difference**	**Limits of agreement**	**Slope of bias**	**Mean difference**	**Limits of agreement**	**Slope of bias**	**Mean difference**	**Limits of agreement**	**Slope of bias**	**Mean difference**	**Limits of agreement**	**Slope of bias**	**Mean difference**	**Limits of agreement**	**Slope of bias**
Rice [2]	1	−80, 82	−0.38***	2	−90, 95	−0.40***	−2	−80, 77	−0.29***	−1	−9, 7	−−−	0	−153, 154	−0.36***
Bread [2]	2	−38, 41	−0.22*	2	−31, 35	−0.55***	0	−9, 9	−0.77***	−1	−8, 6	−−−	2	−51, 55	−0.25*
Noodles [2]	0	−26, 25	−1.01***	−15	−116, 86	−0.75***	4	−46, 54	−0.76***	0	−3, 3	−−−	−11	−130, 108	−0.68***
Miso soup [2]	14	−51, 80	0.04	19	−63, 100	0.42***	16	−60, 92	−0.02	0	−7, 6	−−−	49	−95, 193	0.23**
Potatoes [2]	1	−11, 13	0.05	−5	−34, 24	−1.52***	−4	−39, 31	−1.23***	−1	−8, 6	−−−	−9	−59, 41	−0.89***
Pulses and nuts [2]	−4	−55, 47	−1.01***	−2	−43, 38	−1.20***	−5	−62, 52	−1.13***	−5	−37, 27	−−−	−16	−110, 77	−0.93***
Vegetables [2]	5	−57, 67	−0.29**	−16	−103, 71	−0.53***	−23	−143, 97	−0.47***	−2	−17, 13	−−−	−37	−234, 161	−0.41***
Pickled vegetables	2	−7, 11	0.49***	1	−9, 11	0.14	1	−17, 19	−0.70***	0	0, 0	−−−	4	−21, 29	0.08
Fruit	−6	−61, 49	−0.46***	−4	−52, 43	−0.82***	3	−33, 40	−0.06	6	−41, 54	−0.15	0	−106, 106	−0.23*
Fish and shellfish [2]	−2	−21, 17	−0.96***	−4	−35, 26	−1.04***	−9	−50, 32	−0.87***	0	−6, 5	−−−	−16	−72, 41	−0.78***
Meat [2]	1	−18, 19	−0.58***	−9	−45, 28	−0.84***	−17	−74, 41	−1.06***	0	−4, 4	−−−	−26	−98, 47	−0.73***
Eggs [2]	0	−26, 26	−0.43***	−3	−27, 20	−0.90***	−3	−22, 17	−1.07***	−1	−5, 4	−−−	−7	−47, 33	−0.48***
Dairy products	3	−105, 111	−0.52***	−2	−76, 72	−0.70***	3	−28, 34	−0.02	−8	−91, 74	−0.39***	−5	−187, 177	−0.31**
Confectioneries	1	−29, 32	−0.26	−1	−37, 36	−0.25	2	−14, 17	0.46***	27	−32, 85	0.76***	29	−47, 106	0.51***
Fruit and vegetable juice	5	−37, 47	0.27**	0	−38, 37	−0.59***	2	−19, 24	0.58***	9	−48, 67	0.25*	16	−85, 118	0.47***
Alcoholic beverages [3]	0	−1, 1	−−−	1	−17, 18	1.93***	−19	−181, 143	−0.32***	4	−118, 126	0.14	−15	−211, 182	−0.21***
Soft drinks	5	−35, 45	1.47***	0	−34, 35	−0.26**	1	−44, 46	−0.01	−8	−121, 104	−0.49***	−2	−158, 155	0.18
Green tea	16	−89, 121	0.20*	7	−110, 124	0.20	10	−111, 131	0.01	19	−271, 309	−0.12	51	−441, 543	0.21*
Barley tea	23	−105, 151	−0.03	30	−100, 159	0.05	24	−121, 168	−0.15	45	−299, 388	−0.22*	121	−418, 659	−0.04
Oolong tea	7	−50, 64	1.99***	9	−67, 84	1.09***	4	−51, 58	−0.06	17	−120, 155	0.57***	36	−236, 308	0.83***
Black tea	3	−43, 49	0.00	3	−51, 57	0.37***	4	−30, 38	0.42***	14	−117, 145	0.14	24	−152, 200	0.39***
Coffee	34	−109, 177	−0.10	26	−94, 145	0.30**	9	−66, 84	0.9***	54	−154, 263	0.19*	123	−220, 466	0.30***
Water	55	−85, 195	0.98***	57	−91, 205	1.46***	61	−102, 224	1.66***	53	−395, 500	−0.44***	225	−395, 846	0.31**
Breakfast cereals [4]	2	−10, 15	0.81***	0	−4, 3	−−−	0	−5, 5	−−−	0	0, 0	−−−	2	−12, 16	0.68***

[1] Mean difference was calculated as mean of difference between the two methods (MDHQ minus DR). Limits of agreement were calculated as the mean difference ± 1.96 standard deviation. Slope of bias (regression coefficient) was calculated by using a linear regression analysis, in which the difference between the two methods (MDHQ minus DR) was the dependent variable and the average of the two methods was the independent variable; positive values indicate that the difference increases as the average intake increases, while negative values indicate that the difference decreases as the average intake increases (* p <0.05, ** p <0.01, *** p <0.001). For the food groups the intake of which was not assessed in the MDHQ and thus the intake value was 0 g/day for all participants, the slope of bias was not calculated. [2] Not assessed for snacks in the MDHQ. [3] Not assessed for breakfast in the MDHQ. [4] Only assessed for breakfast in the MDHQ.

Table S11. Bland–Altman analysis for estimates of daily intakes of Tier 1 food groups (in grams per day) derived from the 4-day weighed dietary record (DR) and those derived from the paper version of the Meal-based Diet History Questionnaire (MDHQ) in 111 Japanese men, according to meal type [1].

	Breakfast			Lunch			Dinner			Snacks			Overall diet		
**Tier 1 food group**	**Mean difference**	**Limits of agreement**	**Slope of bias**	**Mean difference**	**Limits of agreement**	**Slope of bias**	**Mean difference**	**Limits of agreement**	**Slope of bias**	**Mean difference**	**Limits of agreement**	**Slope of bias**	**Mean difference**	**Limits of agreement**	**Slope of bias**
Rice [2]	2	−81, 84	−0.19**	4	−119, 126	−0.35***	−14	−139, 112	−0.37***	0	−5, 4	---	−9	−240, 222	−0.39***
Bread [2]	1	−39, 41	−0.08	3	−37, 43	−0.06	−1	−9, 8	−1.05***	−2	−14, 10	---	2	−55, 58	−0.06
Noodles [2]	−1	−37, 34	−1.21***	−43	−174, 88	−0.99***	1	−75, 77	−1.02***	−1	−9, 8	---	−44	−205, 117	−1.02***
Miso soup [2]	6	−75, 87	−0.08	17	−74, 109	0.58***	10	−79, 98	−0.17*	0	0, 0	---	34	−133, 200	0.09
Potatoes [2]	0	−16, 16	−0.35*	−6	−34, 22	−1.38***	−9	−59, 42	−1.50***	−1	−4, 3	---	−15	−80, 50	−1.08***
Pulses and nuts [2]	−4	−48, 40	−0.86***	0	−28, 27	−1.09***	−10	−63, 43	−0.92***	−1	−8, 6	---	−16	−98, 66	−0.87***
Vegetables [2]	−6	−76, 64	−0.59***	−17	−94, 60	−0.33**	−47	−206, 111	−0.60***	−2	−21, 17	---	−72	−289, 146	−0.42***
Pickled vegetables	2	−10, 14	0.58***	2	−11, 15	0.34*	2	−20, 25	−0.80***	0	−3, 2	---	6	−30, 42	−0.08
Fruit	−8	−62, 46	−0.60***	−2	−40, 35	−0.85***	3	−34, 40	0.06	7	−50, 64	−0.50***	0	−101, 102	−0.29**
Fish and shellfish [2]	−1	−21, 18	−0.90***	−3	−35, 29	−0.96***	−15	−78, 47	−1.22***	−1	−11, 9	---	−21	−95, 53	−0.94***
Meat [2]	−2	−29, 25	−0.80***	−10	−51, 31	−0.83***	−30	−111, 52	−1.45***	−1	−5, 4	---	−42	−137, 53	−1.03***
Eggs [2]	−2	−28, 24	−0.32***	−6	−31, 19	−0.79***	−3	−25, 19	−0.95***	−1	−5, 4	---	−12	−53, 29	−0.37***
Dairy products	−11	−128, 106	−0.55***	−5	−63, 54	−0.79***	−4	−50, 43	−0.77***	−12	−94, 70	−0.74***	−31	−203, 141	−0.53***
Confectioneries	−3	−41, 35	−0.40**	−2	−37, 33	−0.74***	1	−23, 26	0.27	17	−40, 74	0.54***	13	−72, 99	0.26*
Fruit and vegetable juice	8	−60, 76	0.42***	2	−35, 40	0.31**	3	−27, 33	0.05	17	−73, 106	0.78***	30	−98, 157	0.28***
Alcoholic beverages [3]	−1	−7, 5	---	0	−62, 61	−0.41***	2	−357, 362	−0.19**	4	−234, 242	−0.23***	6	−340, 352	−0.12*
Soft drinks	5	−44, 53	0.94***	3	−89, 94	−0.50***	0	−115, 116	−1.35***	−19	−259, 221	−0.65***	−11	−355, 332	−0.56***
Green tea	7	−82, 96	−0.10	11	−143, 164	0.12	7	−115, 129	−0.02	21	−277, 318	−0.11	45	−428, 518	0.10
Barley tea	14	−90, 117	0.28**	0	−197, 197	−0.44***	19	−135, 174	−0.01	33	−302, 368	−0.19*	66	−453, 585	0.02
Oolong tea	9	−53, 72	1.10***	2	−61, 66	0.05	4	−73, 81	−0.07	21	−186, 227	0.16	37	−270, 344	0.23**
Black tea	7	−49, 63	0.96***	5	−38, 47	0.99***	2	−33, 36	0.67***	13	−163, 189	0.79***	27	−227, 281	0.99***
Coffee	28	−126, 182	−0.09	35	−88, 159	0.61***	11	−66, 89	0.62***	44	−284, 371	−0.10	118	−328, 564	0.20*
Water	39	−112, 190	0.89***	53	−110, 216	0.97***	66	−102, 234	1.08***	16	−621, 653	−0.72***	175	−541, 891	−0.05
Breakfast cereals [4]	2	−14, 18	0.86***	0	0, 0	---	0	0, 0	---	0	0, 0	---	2	−14, 17	0.87***

[1] Mean difference was calculated as mean of difference between the two methods (MDHQ minus DR). Limits of agreement were calculated as the mean difference ± 1.96 standard deviation. Slope of bias (regression coefficient) was calculated by using a linear regression analysis, in which the difference between the two methods (MDHQ minus DR) was the dependent variable and the average of the two methods was the independent variable; positive values indicate that the difference increases as the average intake increases, while negative values indicate that the difference decreases as the average intake increases (* $p < 0.05$, ** $p < 0.01$, *** $p < 0.001$). For the food groups the intake of which was not assessed in the MDHQ and thus the intake value was 0 g/day for all participants, the slope of bias was not calculated. [2] Not assessed for snacks in the MDHQ. [3] Not assessed for breakfast in the MDHQ. [4] Only assessed for breakfast in the MDHQ.

Table S12. Estimates of overall intakes of Tier 2 food groups (in grams per day) derived from the 4-day weighed dietary record (DR) and those derived from the paper version of the Meal-based Diet History Questionnaire (MDHQ), Spearman correlation coefficients, and Bland–Altman analysis in 111 Japanese women and 111 Japanese men [1].

	Women						Men					
				Bland–Altman analysis [4]						Bland–Altman analysis [2]		
Tier 2 food group	Median (P25, P75) [1]	Correlation [2]	Mean ± SD [3]	Mean difference	Limits of agreement	Slope of bias	Median (P25, P75)	Correlation [2]	Mean ± SD	Mean difference	Limits of agreement	Slope of bias
Rice												
White rice	211 (147, 252)	0.62***	199 ± 80	−7	−167, 153	−0.37***	301 (240, 375)	0.57***	297 ± 107*	−23	−252, 206	−0.37***
Brown rice	0 (0, 7)***	0.37***	10 ± 33**	7	−48, 62	0.75***	0 (0, 7)***	0.39***	16 ± 43***	14	−58, 87	1.23***
Bread												
White bread	29 (12, 46)	0.57***	31 ± 21	−4	−57, 49	−0.47***	27 (7, 52)	0.58***	33 ± 28	−2	−62, 57	−0.23*
Wholegrain bread	5 (0, 12)***	0.10	7 ± 8***	6	−11, 23	0.89***	0 (0, 4)***	−0.09	4 ± 12**	4	−21, 29	1.47***
Noodles												
Wheat noodles	25 (12, 41)***	0.19*	30 ± 23***	13	−63, 89	−0.65***	25 (11, 38)**	0.25**	28 ± 25	2	−104, 108	−1.12***
Chinese noodles	7 (1, 16)	0.30**	11 ± 13*	−7	−74, 59	−1.25***	12 (3, 23)	0.33***	17 ± 19*	−9	−96, 79	−1.18***
Instant noodles	0 (0, 4)*	0.38***	4 ± 11***	−8	−56, 40	−1.06***	3 (0, 6)	0.46***	5 ± 10***	−25	−119, 70	−1.61***
Spaghetti	6 (1, 10)	0.17	8 ± 12**	−7	−54, 39	−0.96***	5 (1, 9)	0.26**	7 ± 11**	−9	−66, 48	−1.28***
Buckwheat noodles	1 (0, 7)***	0.35***	5 ± 8	−1	−36, 34	−1.05***	1 (0, 6)***	0.28**	4 ± 7	−4	−46, 39	−1.42***
Pulses and nuts												
Soy milk	0 (0, 0)	0.56***	1 ± 3***	−13	−91, 65	−1.86***	0 (0, 0)	0.40***	0 ± 1	−2	−30, 25	−1.87***
Tofu (i.e., soybean curd)	27 (15, 45)**	0.39***	31 ± 20*	6	−47, 59	−0.40**	24 (13, 36)	0.43***	27 ± 18	−4	−71, 63	−0.94***
Natto (i.e., fermented soybeans)	4 (1, 8)*	0.53***	6 ± 6**	−2	−19, 15	−0.68***	2 (0, 6)	0.66***	5 ± 10	−1	−16, 14	−0.06
Tofu products	3 (1, 6)*	0.31***	4 ± 4	0	−15, 15	−1.02***	2 (0, 6)	0.28**	3 ± 4*	−3	−25, 19	−1.40***
Peanuts and nuts	0 (0, 0)***	0.28**	0 ± 0***	−5	−29, 19	−1.94***	0 (0, 0)***	0.37***	0 ± 1***	−5	−22, 13	−1.85***
All other pulses and nuts	0 (0, 0)	0.18	0 ± 0***	−2	−12, 8	−1.97***	0 (0, 0)	0.11	1 ± 1*	−1	−12, 9	−1.86***
Vegetables												
Edamame (i.e., immature soybeans) and peas	0 (0, 2)	0.24**	1 ± 2	−1	−17, 15	−1.70***	0 (0, 2)	0.33***	2 ± 2	−1	−11, 10	−1.12***
Seaweeds	5 (2, 9)	0.20*	7 ± 6	0	−17, 17	−0.38*	4 (1, 8)	0.33***	6 ± 6	−1	−22, 21	−0.84***
Pumpkins	3 (0, 5)	0.41***	4 ± 4**	−3	−23, 17	−1.18***	3 (0, 5)	0.48***	4 ± 4***	−4	−27, 19	−1.23***
Mushrooms	7 (3, 14)*	0.45***	10 ± 8**	−3	−27, 21	−0.72***	7 (2, 13)***	0.46***	9 ± 7***	−5	−28, 18	−0.74***
Cabbage	16 (10, 29)*	0.39***	20 ± 14**	−7	−53, 40	−0.82***	17 (8, 29)***	0.37***	22 ± 19***	−13	−70, 45	−0.62***
Cucumbers	10 (5, 17)	0.50***	11 ± 8	−2	−27, 23	−0.76***	9 (5, 16)	0.49***	11 ± 8	−2	−25, 22	−0.61***
Bitter melon	0 (0, 1)	0.46***	1 ± 2**	−2	−14, 10	−1.44***	0 (0, 1)	0.53***	1 ± 2*	−1	−11, 9	−1.33***
Burdock	0 (0, 1)**	0.43***	1 ± 1***	−2	−10, 7	−1.64***	0 (0, 2)***	0.25**	1 ± 1***	−3	−15, 9	−1.72***
Radishes	9 (2, 16)	0.39***	11 ± 11	−1	−31, 30	−0.48***	9 (2, 17)	0.23*	11 ± 11	−1	−34, 32	−0.57***
Onions	27 (15, 40)	0.19*	29 ± 20	2	−49, 53	−0.11	23 (12, 39)	0.22*	26 ± 19	−7	−80, 67	−1.01***
Chinese cabbage	1 (0, 2)	0.38***	2 ± 3	−1	−13, 11	−1.02***	1 (0, 4)*	0.20*	3 ± 5	0	−16, 16	−0.72***
Tomatoes	18 (6, 36)***	0.49***	21 ± 17**	5	−34, 45	−0.22	17 (4, 30)*	0.39***	19 ± 16	1	−70, 72	−1.12***
Eggplants	6 (3, 11)	0.47***	8 ± 6	−2	−33, 29	−1.20***	6 (2, 10)	0.50***	7 ± 6*	−4	−45, 37	−1.37***
Carrots	15 (9, 26)***	0.51***	18 ± 13***	5	−22, 31	−0.09	14 (4, 22)	0.40***	16 ± 14	2	−29, 33	−0.04
Green peppers	3 (1, 6)	0.44***	4 ± 3*	−2	−16, 12	−1.19***	3 (1, 5)**	0.41***	4 ± 3***	−3	−21, 14	−1.30***
Broccoli	2 (0, 4)	0.43***	3 ± 3	−2	−18, 15	−1.26***	2 (0, 4)	0.47***	2 ± 3**	−2	−18, 14	−1.24***

Table S12. Continued

Tier 2 food group	Women						Men					
				Bland–Altman analysis [4]						Bland–Altman analysis [2]		
	Median (P25, P75) [1]	Correlation [2]	Mean ± SD [3]	Mean difference	Limits of agreement	Slope of bias	Median (P25, P75)	Correlation [2]	Mean ± SD	Mean difference	Limits of agreement	Slope of bias
Green leafy vegetables	15 (5, 26)**	0.44***	17 ± 14	2	−40, 45	−0.59***	14 (3, 22)	0.23*	16 ± 14	2	−35, 40	−1.24
Bean sprouts	4 (2, 6)***	0.37***	4 ± 4***	−6	−31, 19	−1.43***	4 (1, 7)***	0.34***	5 ± 5***	−8	−36, 20	−1.30***
Lettuce	4 (1, 7)	0.45***	5 ± 5*	−2	−19, 15	−0.91***	4 (1, 8)	0.46***	6 ± 6	−2	−35, 30	−1.27***
All other vegetables	1 (1, 2)***	0.02	1 ± 1***	−18	−55, 20	−2.00***	1 (1, 2)***	0.03	1 ± 1***	−20	−62, 21	−2.00***
Fruit												
Strawberries	0 (0, 0)**	−0.01	0 ± 1	0	−2, 2	1.87***	0 (0, 0)**	−0.05	1 ± 2*	0	−3, 4	1.92***
Persimmons	0 (0, 0)**	0.34***	1 ± 2	0	−5, 5	0.10	0 (0, 0)	0.37***	1 ± 4	0	−9, 10	−0.12**
Citrus	4 (0, 13)***	0.14	11 ± 19***	6	−25, 36	0.48***	0 (0, 9)***	0.24*	8 ± 13***	5	−20, 29	0.45***
Kiwi fruit	0 (0, 2)	0.51***	1 ± 3	0	−10, 9	−0.74***	0 (0, 1)	0.50***	1 ± 2*	−1	−10, 8	−1.13***
Watermelon	0 (0, 2)***	0.17	1 ± 3	0	−21, 20	−1.60***	0 (0, 1)***	0.15	1 ± 2	0	−13, 12	−1.60***
Pears	5 (1, 13)	0.54***	11 ± 15	−1	−38, 36	−0.37**	1 (0, 9)	0.48***	7 ± 12	−4	−44, 37	−0.87***
Bananas	5 (0, 22)***	0.65***	16 ± 24*	4	−37, 45	0.16	1 (0, 13)	0.65***	14 ± 27	1	−40, 42	0.03
Grapes	3 (1, 9)	0.45***	9 ± 15*	−7	−59, 46	−0.92***	1 (0, 8)	0.49***	7 ± 15	−5	−61, 51	−0.94***
Melon	0 (0, 0)***	0.22*	1 ± 2	−1	−30, 27	−1.90***	0 (0, 0)***	0.13	1 ± 2*	1	−4, 5	0.87***
Peaches	0 (0, 1)**	0.09	1 ± 3	−1	−18, 17	−1.44***	0 (0, 0)	0.23*	1 ± 3	−1	−22, 20	−1.54***
Apples	3 (0, 18)***	0.46***	12 ± 19***	6	−25, 36	0.28**	0 (0, 12)***	0.44***	9 ± 19***	5	−22, 31	0.42***
All other fruit	1 (0, 1)	0.15	1 ± 1**	−9	−68, 50	−1.98***	1 (0, 2)	0.06	2 ± 4	−2	−25, 21	−1.46***
Fish and shellfish												
Oily fish	6 (1, 11)	0.19*	7 ± 6*	−4	−42, 34	−1.45***	8 (2, 13)*	0.29**	8 ± 7**	−6	−42, 31	−1.31***
Red meat fish	3 (1, 6)	0.08	4 ± 4	−1	−17, 16	−1.06***	5 (1, 9)*	0.16	6 ± 6	1	−21, 23	−0.78***
Squid and octopus	0 (0, 2)	0.28**	1 ± 2*	−1	−11, 9	−1.42***	1 (0, 4)	0.37***	2 ± 3**	−2	−15, 12	−1.28***
Eel	0 (0, 0)***	0.24*	0 ± 0	0	−3, 3	−1.81***	0 (0, 0)***	0.26**	0 ± 1	−1	−9, 7	−1.84***
Shrimp and crab	0 (0, 2)	0.06	1 ± 2**	−1	−11, 9	−1.55***	1 (0, 3)	0.08	2 ± 3	−2	−17, 13	−1.21***
Shellfish	0 (0, 2)	0.13	1 ± 1	0	−7, 6	−1.34***	0 (0, 2)*	0.24*	1 ± 2	0	−7, 7	−0.99***
Small fish with bones	0 (0, 1)**	0.26**	1 ± 1	0	−4, 3	−0.55***	0 (0, 0)**	0.33***	0 ± 1	0	−5, 5	−1.58***
Fish eggs	0 (0, 0)	0.25**	0 ± 1**	−1	−6, 4	−1.68***	0 (0, 0)	0.31***	0 ± 0**	−1	−7, 5	−1.90***
Dried fish	1 (0, 3)	0.28**	2 ± 3	−2	−21, 18	−1.50***	1 (0, 4)	0.24*	3 ± 4*	−3	−27, 22	−1.44***
Salmon	3 (1, 5)*	0.29**	4 ± 5***	−4	−26, 19	−1.22***	4 (1, 8)*	0.15	5 ± 4***	−5	−31, 22	−1.49***
White meat fish	1 (1, 7)	0.27**	4 ± 5	−1	−23, 21	−1.19***	5 (1, 10)	0.22*	6 ± 6	−1	−26, 25	−1.18***
Ground fish meat products	4 (1, 7)*	0.26**	5 ± 5	0	−15, 16	−0.68***	5 (1, 8)	0.34***	6 ± 6	−2	−30, 26	−1.13***
Canned tuna	1 (0, 1)	0.39***	1 ± 1**	−1	−7, 5	−1.31***	0 (0, 1)	0.24*	1 ± 3*	−1	−10, 8	−0.76***
All other fish and shellfish	0 (0, 0)***	0.10	0 ± 0	0	0, 0	−1.81***	0 (0, 0)***	−0.15	0 ± 0	0	−2, 2	−2.00***
Meat												
Beef	6 (1, 9)**	0.21*	7 ± 6***	−6	−38, 27	−1.42***	11 (5, 15)	0.16	11 ± 9*	−5	−50, 40	−1.42***
Chicken	17 (11, 22)***	0.34***	17 ± 8***	−14	−62, 34	−1.38***	18 (13, 26)***	0.17	20 ± 9***	−26	−103, 50	−1.69***
Processed meat	8 (4, 14)	0.40***	10 ± 8	0	−21, 20	−0.40**	11 (6, 20)	0.29**	14 ± 11	0	−29, 29	−0.36*
Pork	25 (18, 31)*	0.33***	25 ± 12*	−5	−52, 41	−1.02***	27 (20, 38)***	0.06	29 ± 13***	−10	−69, 49	−1.27***

Table S12. Continued

Tier 2 food group	Women						Men					
				Bland–Altman analysis [4]						Bland–Altman analysis [2]		
	Median (P25, P75) [1]	Correlation [2]	Mean ± SD [3]	Mean difference	Limits of agreement	Slope of bias	Median (P25, P75)	Correlation [2]	Mean ± SD	Mean difference	Limits of agreement	Slope of bias
Liver	0 (0, 0)***	0.18	0 ± 0	0	−5, 5	−1.92***	0 (0, 0)***	0.21*	0 ± 0	0	−8, 7	−1.92***
All other meat	0 (0, 0)***	0.01	0 ± 0	0	−3, 3	−2.00***	0 (0, 0)***	−0.12	0 ± 0	0	−5, 5	−2.00***
Dairy products												
Ice cream	1 (0, 4)	0.36***	4 ± 8	−2	−28, 24	−0.77***	1 (0, 4)	0.30**	3 ± 5	−3	−31, 25	−1.34
Cheese	1 (0, 4)**	0.25**	4 ± 10	−1	−24, 21	0.77***	1 (0, 4)*	0.21*	3 ± 6*	−2	−18, 14	−0.21
Low-fat milk	0 (0, 0)	0.51***	5 ± 17*	−5	−54, 44	−0.77***	0 (0, 0)	0.62***	9 ± 31	−9	−123, 105	−0.95***
Yogurt	19 (1, 59)**	0.60***	44 ± 63**	−24	−178, 131	−0.50***	2 (0, 17)***	0.64***	27 ± 53***	−26	−144, 93	−0.53***
Full-fat milk	60 (14, 107)***	0.61***	68 ± 60***	30	−88, 148	0.08	7 (0, 57)***	0.56***	32 ± 45**	12	−72, 95	0.09
All other dairy products	0 (0, 1)	−0.08	1 ± 2**	−3	−24, 18	−1.87***	0 (0, 1)	−0.03	2 ± 4	−4	−43, 36	−1.83***
Confectioneries												
Candies, caramels, and chewing gum	0 (0, 1)***	0.20*	1 ± 1	0	−4, 4	−0.48**	0 (0, 0)***	0.28**	1 ± 2***	1	−3, 4	1.12***
Japanese bread with a sweet filling	3 (1, 8)***	0.24*	6 ± 7*	3	−18, 23	−0.38*	1 (0, 6)	0.37***	7 ± 13	−1	−34, 32	−0.13
Snacks made from wheat flour	1 (0, 4)***	0.46***	3 ± 4***	2	−7, 10	0.04	1 (0, 4)***	0.34***	3 ± 5	1	−11, 13	−0.55***
Jellies	0 (0, 1)***	0.13	1 ± 2**	−3	−24, 18	−1.92***	0 (0, 0)***	0.16	0 ± 1**	−4	−31, 23	−1.94***
Rice crackers	5 (1, 10)***	0.43***	7 ± 9***	5	−10, 21	0.86***	3 (0, 9)***	0.46***	7 ± 11***	5	−14, 25	1.31***
Chocolates	7 (2, 16)***	0.43***	12 ± 16***	8	−21, 37	1.16***	2 (0, 14)***	0.41***	9 ± 13***	6	−16, 28	1.13***
Biscuits and cookies	2 (0, 6)***	0.35***	4 ± 5***	2	−8, 12	0.05	0 (0, 4)***	0.39***	2 ± 4*	1	−7, 9	0.15
Cakes	14 (3, 26)***	0.42***	18 ± 19***	9	−31, 49	0.20	6 (1, 18)***	0.31**	12 ± 16*	4	−34, 42	−0.07
Japanese sweets	8 (2, 19)**	0.23*	12 ± 14*	4	−29, 37	−0.03	2 (0, 12)*	0.52***	7 ± 11	0	−31, 31	−0.54***
Alcoholic beverages												
Beer	0 (0, 25)	0.78***	50 ± 127	−6	−110, 99	−0.10*	50 (0, 206)	0.80***	185 ± 307	11	−250, 272	−0.07
Sake	0 (0, 0)	0.64***	2 ± 13	1	−9, 10	0.32***	0 (0, 0)	0.60***	9 ± 27	2	−41, 45	−0.30***
Shochu (i.e., Japanese distilled beverages)	0 (0, 0)	0.70***	20 ± 66	−4	−98, 90	−0.05	2 (0, 67)	0.72***	62 ± 108	−3	−210, 204	−0.40***
Wine	0 (0, 0)	0.48***	6 ± 22	−3	−90, 83	−1.00***	0 (0, 0)	0.58***	5 ± 20	−1	−34, 32	−0.48***
Whiskey and other spirits	0 (0, 0)	—– [5]	0 ± 2	0	−3, 3	2.00***	0 (0, 0)	0.90***	7 ± 31	1	−39, 40	0.16*

P25, 25th percentile; P75, 75th percentile; SD, standard deviation. [1] The values derived from the MDHQ were compared with those derived from the DR (shown in Table 5) using the Wilcoxon signed-rank test: * p <0.05, ** p <0.01, *** p <0.001. [2] Spearman correlation coefficients: * p <0.05, ** p <0.01, *** p <0.001. [3] The values derived from the MDHQ were compared with those derived from DR (shown in Table S6) using the paired t-test: * p <0.05, ** p <0.01, *** p <0.001. [4] Mean difference was calculated as mean of difference between the two methods (MDHQ minus DR). Limits of agreement were calculated as the mean difference ± 1.96 SD. Slope of bias (regression coefficient) was calculated by using a linear regression analysis, in which the difference between the two methods (MDHQ minus DR) was the dependent variable and the average of the two methods was the independent variable; positive values indicate that the difference increases as the average intake increases, while negative values indicate that the difference decreases as the average intake increases (* p <0.05, ** p <0.01, *** p <0.001). [5] Not available because the intake derived from the DR was 0 g/day for all participants.
